# Inducible displacement measured with radiostereometric analysis in primary knee arthroplasty: a systematic review of clinical studies

**DOI:** 10.2340/17453674.2026.46318

**Published:** 2026-09-23

**Authors:** Jonathan Hugo JÜRGENS-LAHNSTEIN, Johanne Frost TEILMANN, Emil Toft PETERSEN, Søren RYTTER, Maiken STILLING, Elise LAENDE

**Affiliations:** 1 Department of Clinical Medicine, Aarhus University, Aarhus, Denmark; 2 AutoRSA Research Group, Aarhus, Denmark; 3 Department of Orthopedic Surgery, Aarhus University Hospital, Aarhus, Denmark; 4 Systems Design Engineering, University of Waterloo, Canada

## Abstract

**Background and purpose:**

Radiostereometric analysis (RSA) is the gold standard for assessing implant migration, with 1-year migration thresholds predicting later revision. While inducible displacement under immediate load may enable single-session functional stability assessment, no established threshold exists. We systematically reviewed RSA-measured inducible displacement after primary knee arthroplasty to determine whether a threshold identifying at-risk implants can be derived from the existing literature, and to characterize responses to loading, differences between fixation methods, temporal patterns, and the relationship to migration.

**Methods:**

We searched PubMed, Web of Science, Scopus, and Embase (April 2025) for studies reporting in vivo RSA-measured inducible displacement after primary knee arthroplasty, requiring quantitative data with specified reference and load conditions. Studies were evaluated for links between inducible displacement and supine, non-weightbearing migration. We assessed bias risk using RoB 2 and ROBINS-E. Due to high heterogeneity, a descriptive synthesis was performed; meta-analysis and regression were not feasible (PROSPERO CRD420251043748).

**Results:**

23 studies were included. The most common outcome was maximum total point motion (MTPM) during supine-to-single-leg weightbearing (SLWB). Cemented tibial components tended to show greater displacement than cementless designs. Rotatory stress tests produced the largest absolute displacements; differences within the same study from SLWB were minimal. Predictive evidence remained limited.

**Conclusion:**

Inducible displacement may indicate functional stability. The existing literature provides no threshold to identify at-risk implants and reporting was too heterogeneous to support a cut-off value. SLWB was the most common protocol. Prospective studies with standardized protocols and clinical endpoints are necessary.

Radiostereometric analysis (RSA), developed by Selvik in the 1970s, is a precise method for evaluating the 3-dimensional micromotion of implants using stereo radiographs [1]. With high precision, RSA enables detection of clinically significant implant migration using small sample sizes, typically 15–25 patients per group [2–4]. Micromotion of an implant with respect to the periprosthetic bone measured over time is defined as migration, whereas instantaneous micromotion of an implant occurring in response to external forces is termed inducible displacement [5]. RSA has been recommended for the phased introduction of new implants [6–8].

Risk stratification models that use early migration patterns can predict long-term outcomes [9]. A limitation of conventional RSA-measured migration is the need for multiple follow-up examinations. Inducible displacement implant motion in response to loading, such as differences between supine and weightbearing conditions, was first studied by Ryd et al. in 1986 [5]. Since then, inducible displacement has been reported in various studies, but with heterogeneous methods and without clear thresholds for clinical relevance. If validated, inducible displacement could serve as a functional stability metric, potentially reducing the need for long-term follow-up by identifying at-risk implants earlier, possibly within a single examination session. CT-based RSA (CT-RSA), which eliminates the need for bone marker insertion, has increased interest in evaluating inducible displacement, particularly with weightbearing CT in symptomatic patients [10].

Guidelines for RSA studies have recently been introduced to improve transparency and consistency of execution [11]. This review aimed to synthesize RSA-measured inducible displacement in primary knee arthroplasty. Our primary aim was to determine whether a threshold identifying at-risk implants can be derived from the existing literature. Our secondary aims were to characterize reported values, responses to loading, differences between fixation methods, temporal patterns, and the relationship to migration.

## Methods

### Study design

PubMed, Web of Science, Scopus, and Embase were searched on April 30, 2025, using the following string (adapted as per database): “Arthroplasty, Replacement, Knee” [MeSH] AND (“Radiostereometric Analysis” [MeSH] OR “radiostereometric analysis” OR Radiostereometric analysis OR “RSA” OR RSA OR “radiostereometric measurements”) AND (inducible micromotion OR inducible migration OR inducible displacement) (Search Strategy, see Supplementary data). Two researchers independently screened titles, abstracts, and full texts, Discrepancies were resolved by a third reviewer (EL). Covidence (https://www.covidence.org/) managed deduplication and screening.

The study is reported according to PRISMA 2020 guidelines.

### Eligibility criteria

Studies were included if they reported RSA-measured inducible displacement in primary knee arthroplasties in humans, providing follow-up duration, quantitative displacement values (MTPM, translations, or rotations), and a description of both the reference and loaded examinations. Studies were required to report micromotion using a right-handed coordinate system, with left-sided data mirrored to right-sided anatomy in accordance with international standardization guidelines, using either a local implant or calibration box/cage coordinate system, provided the knee was aligned with the calibration box. Excluded were non-peer-reviewed publications, as these lack formal quality assurance, and studies without quantitative displacement values, as these could not be included in the evidence synthesis. Only English-language articles were included, as translation resources were not available. No date restrictions were applied, so as to capture the full historical development of inducible displacement research.

### Data extraction

All pre-specified PROSPERO outcomes were sought during extraction. A meta-analysis of inducible displacement, a regression analysis correlating migration with inducible displacement, and the definition of clinical cut-offs could not be synthesized as planned, as detailed in the “Deviations from protocol” section. One researcher extracted data from each study into Excel, collecting: RSA and inducible displacement examination time points, prosthesis type (name, total knee arthroplasty [TKA] or unicompartmental knee arthroplasty [UKA]), fixation method (cemented or cementless), and displacement values (MTPM, translations, and rotations) as mean, median, range (min–max), and standard deviation (SD) or 95% confidence interval (CI) where available. Original authors were not contacted for missing or unextractable data, for pre-2000 studies contact was considered infeasible, and for more recent studies, missing data points did not alter the overall descriptive conclusions.

### Study risk of bias assessment

Risk of bias was assessed by two reviewers using RoB 2 and ROBINS-E; disagreements were resolved by consensus [12,13].

### Reporting bias assessment

Funnel plot analysis was not performed, as the absence of pooled effect estimates rendered it inappropriate. Reporting bias remains a potential limitation, as null or inconclusive findings on inducible displacement may be underrepresented in the published literature.

### Certainty assessment

GRADE assessment was not performed. Given the descriptive synthesis, substantial methodological heterogeneity, absence of pooled effect estimates, and lack of a defined outcome threshold, formal certainty grading was considered uninformative. The overall certainty of evidence is addressed narratively in the “Limitations” section.

### Deviations from protocol

Due to the high degree of methodological heterogeneity across the 23 included studies, specifically regarding loading protocols, follow-up durations, and implant designs, the following deviations from the original protocol were made:

1. ***Omission of meta-analysis***: For purely descriptive purposes only, sample-size-weighted means were computed where outcomes were approximately comparable. These figures are illustrative and do not constitute pooled estimates.
2. ***Omission of regression analysis***: The planned regression analysis to correlate supine RSA migration with weightbearing inducible displacement was not performed due to the limited number of studies reporting both values at identical time points.
3. ***Threshold definition***: The objective of defining clinically relevant cut-off values for inducible displacement was omitted, as inconsistent reporting and small sample sizes prevented a robust determination of these values.

### Statistics

Statistical analyses were performed using R version 4.5.1 (R Foundation for Statistical Computing, Vienna, Austria) [14]. Due to the significant heterogeneity in loading protocols, follow-up durations, and implant designs, a formal meta-analysis was not performed. Consequently, no summary effect measure was calculated. Findings are presented as observed values from individual studies, with sample-size-weighted descriptive means computed for illustrative purposes only where outcomes were approximately comparable. These descriptive summaries do not constitute effect estimates and carry no inferential weight. Instead, we conducted a descriptive synthesis of evidence. SDs were extracted directly from the articles or estimated from reported ranges [15]. Heterogeneity across included studies was assessed qualitatively by comparing loading protocols, follow-up durations, implant designs, and reporting formats. Statistical assessment of heterogeneity (e.g., I² statistic) was not performed because the absence of a formal meta-analysis rendered such measures inappropriate. The degree of methodological heterogeneity observed across the 23 included studies was a primary reason for conducting a descriptive rather than quantitative synthesis.

### Ethics, registration, data sharing plan, funding, and disclosures

Ethical approval was not required. The protocol was registered in PROSPERO. Due to an administrative error, the PROSPERO entry was first published on September 17, 2025, even though it was created on April 30, 2025. This represents a limitation and potential source of bias, as the protocol was not publicly available during the conduct of the review. Data and the R code used for descriptive analyses are available upon reasonable request from the corresponding author. The protocol was registered on PROSPERO (CRD420251043748) and is publicly accessible at https://www.crd.york.ac.uk/PROSPERO/view/CRD420251043748. This systematic review is part of a PhD project funded by Inger og Asker Larsens Fond, Sofus Carl Emil Frris, Forskningsfond I Aarhus, Guildal Fonden, and Kong Christian d. 10 Fond. The funders had no role in the study design, data analysis, interpretation, or decision to publish. No competing interests are declared. Complete disclosure of interest forms according to ICMJE are available on the article page, doi: 10.2340/17453674.2026.46318

## Results

### Literature search

**Figure 1:**
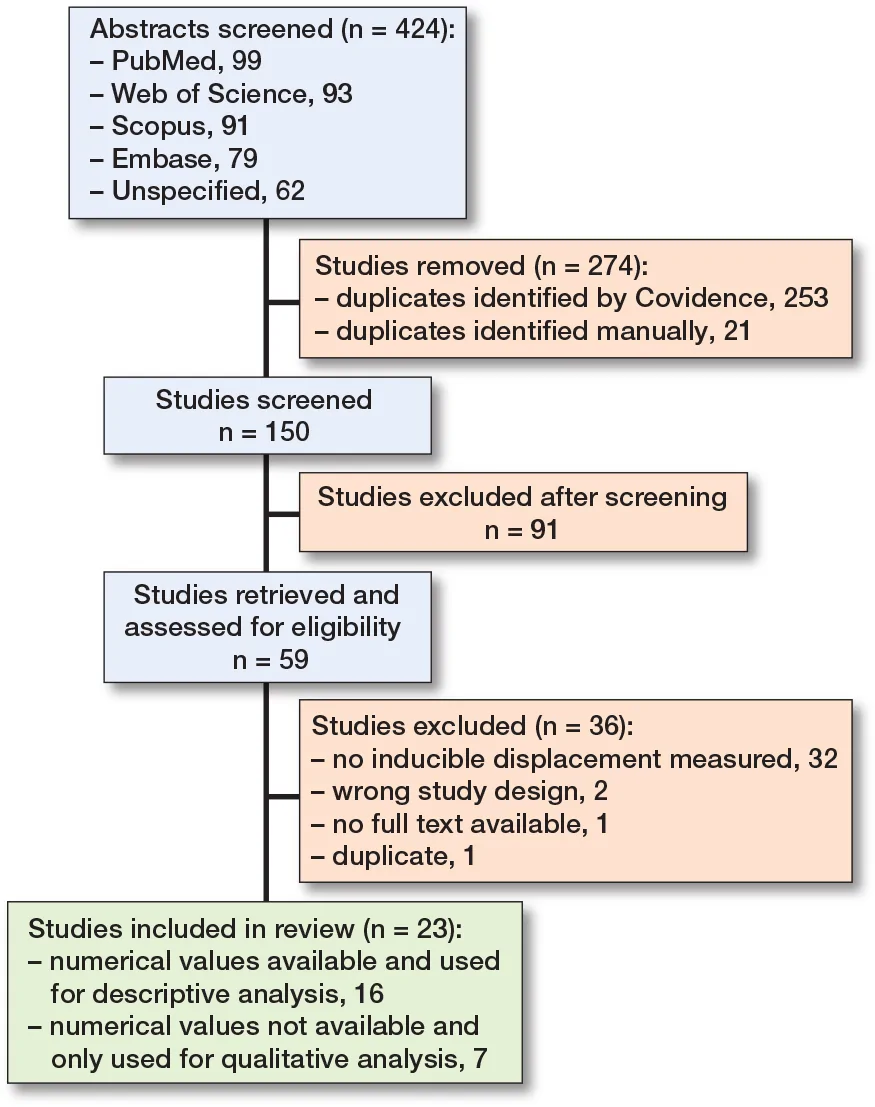
Flow chart of study extraction process.

The literature search identified 424 abstracts. After removing 274 duplicates, 150 abstracts were screened, resulting in 23 studies included for data extraction (Figure 1, Table 1) [5,16-37]. Studies assessed at the full-text level but subsequently excluded are listed with individual reasons in Table A1 (see Supplementary data).

**Table 1:** Articles included

First author, year	Title	Prosthesis	Fixation	UKA/TKA	Used for quantitative analysis	ID
Ryd 1986 [5]	Tibial component fixation in knee arthroplasty	Kinematic Condylar Knee, with metal backed tibial component	Cemented	TKA	No	1
Ryd 1986 [34]	Micromotion of conventionally cemented all-polyethylene tibial components in total knee replacements	Condylar Total Knee	Cemented	TKA	Yes	2
Ryd 1988 [33]	Micromotion of noncemented Freeman–Samuelson knee prostheses in gonarthrosis. A roentgenstereophotogrammetric analysis of 8 successful cases	Freeman–Samuelson	Cementless	TKA	No	3
Ryd 1992 [32]	The influence of metal backing in unicompartmental tibial component fixation. An in vivo roentgen stereophotogrammetric analysis of micromotion	Lund Tibial Component	Cemented	UKA	No	4
Ryd 1993 [31]	Micromotion of a noncemented tibial component with screw fixation. An in vivo roentgen stereo-photogrammetric study of the Miller–Galante prosthesis	Miller-Galante total knee prosthesis	Cementless	TKA	No	5
Hilding 1995 [30]	The stability of 3 different cementless tibial components	PCA Modular	Cementless	TKA	Yes	6
Petersen 1999 [29]	Preoperative bone mineral density of the proximal tibia and migration of the tibial component after uncemented total knee arthroplasty	PCA Modular	Cementless	TKA	No	7
Regner 2000 [23]	Tibial component fixation in porous- and hydroxyapatite-coated total knee arthroplasty: a radiostereometric evaluation of migration and inducible displacement after 5 years	Freeman–Samuelson) hydroxyapatite-coated (FS HA)	Cementless	TKA	Yes	8
Uvehammer 2001[37]	Inducible displacements of cemented tibial components during weightbearing and knee extension. Observations during dynamic radiostereometry related to joint positions and 2 years’ history of migration in 16 TKR	AMK	Cemented	TKA	Yes	9
Hansson 2004 [21]	Mobile vs fixed meniscal bearing in total knee replacement: a randomized radiostereometric study	Rotaglide Total Knee System (RTK) Porous coated	Cementless	TKA	Yes	10
Bragonzoni 2005 [22]	The stress inducible displacement detected through RSA in non-migrating UKR	Duracon UNI	Cemented	UKA	Yes	11
Wilson 2010 [20]	Inducible displacement of a trabecular metal tibial monoblock component	Nexgen LPS Monoblock, uncemented (TM)	Cementless	TKA	Yes	12
Horsager 2017 [19]	Dynamic RSA for the evaluation of inducible micromotion of Oxford UKA during step-up-step down motion	Oxford Unicompartmental, Knee Arthroplasty	Cemented	UKA	No	13
Cheung 2018 [35]	Inducible displacement of cemented tibial components ten years after total knee arthroplasty	Genesis II TKA	Cemented	TKA	Yes	14
Laende 2019 [36]	A randomized controlled trial of tibial component migration with kinematic alignment using patient-specific instrumentation versus mechanical alignment using computer-assisted surgery in total knee arthroplasty	Triathalon total knee	Cemented	TKA	Yes	15
Laende 2019 [18]	Predictive value of short-term migration in determining long-term stable fixation in cemented and cementless total knee arthroplasties	NexGen Option Stemmed	Cemented	TKA	Yes	16
Laende 2020 [17]	Tibial component migration after total knee arthroplasty with high-viscosity bone cement	Triathalon Total Knee	Cemented	TKA	Yes	17
Broberg 2022 [16]	Migration and inducible displacement of the bicruciate-stabilized total knee arthroplasty: a randomized controlled trial of gap balancing and measured resection techniques	Journey II BCS TKA	Cemented	TKA	Yes	18
Broberg 2023 [27]	A multimodal assessment of cementless tibial baseplate fixation using radiography, radiostereo-metric analysis, and magnetic resonance imaging	Triathalon Tritanium	Cementless	TKA	Yes	19
Teeter 2023 [28]	Axial and sagittal rotation of cementless tibial baseplates occurs in bone under joint loading	Triathalon Tritanium	Cementless	TKA	No	20
Turgeon 2024 [26]	Randomized controlled trial comparing traditional versus enhanced-fixation designs of a novel cemented total knee arthroplasty tibial component	Attune Knee System	Cemented	TKA	Yes	21
Puijk 2025 [24]	5-year migration and inducible displacement of the uncemented LCS and ATTUNE rotating platform knee systems: a secondary report of a randomized controlled RSA trial	LCS	Cementless	TKA	Yes	22
Hext 2025 [25]	Inducible displacement of cementless total knee arthroplasty components with conventional and weight-bearing CT-based radiostereometric analysis	Triathalon Tritanium	Cemented	TKA	Yes	23

TKA = total knee arthroplasty; UKA = unicompartmental knee arthroplasty.

### Risk of bias in studies

The overall risk of bias in the included RCTs ranged from low to some concerns, primarily due to missing outcome data (Table A2, see Supplementary data). In the observational studies, risk of bias ranged from low to high, with the selection of reported results being the primary concern (Table A3, see Supplementary data).

### Prostheses

The included studies covered 19 different prosthesis designs (Table 1). No study included both TKA and UKA. 3 studies reported on both cemented and cementless tibial baseplates.

### Inducible displacement stress examinations

Extracted values are presented in Table A4 (see Supplementary data). The studies described 17 distinct examination types and 21 reference-to-displacement combinations (Table 2). The most common approach used supine as the reference and single-leg weightbearing as the loaded condition (18 studies). MTPM was the most frequently reported outcome (19 studies), followed by rotations around the x- and y-axes (5 studies) and z-axis (medial–lateral tilt, 4 studies). Coordinate systems varied across studies; all values were corrected for left knees. The highest and lowest observed values are summarized in Table 3 for descriptive purposes only and are not directly comparable across rows given the heterogeneity of loading protocols and follow-up durations.

**Table 2:** Overview of inducible displacement examinations

Reference examination	Loaded examination	n	Ref
Supine	Weightbearing single leg	18	[5,16–18,20–22,24–27,29–34,36]
Supine	Inward rotatory stress leg on platform—10 Nm	5	[5,20,23,30,33]
Supine	Outward rotatory stress leg on platform—10 Nm	5	[5,20,23,30,33]
Outward rotatory stress leg on platform – 10 nm	Inward rotatory stress leg on platform—10 Nm	2	[30,34]
Standing	Step-up-step-down	2	[19,19]
Supine	Weightbearing both legs	2	[28,35]
Inward rotatory stress leg on platform—10 nm	Outward rotatory stress leg on platform—10 Nm	1	[20]
Outward rotatory stress leg on platform	Inward rotatory stress log on platform	1	[21]
Standing	Inward rotatory stress—only muscle force	1	[35]
Supine	Inward rotatory stress±—only muscle force	1	[35]
Supine	Passive stress test	1	[20]
Supine	Vertical position, hanging knee, non-weightbearing	1	[32]
Supine	Weightbearing single leg—flexed 45–60°	1	[30]
Weightbearing single leg	Inward rotatory stress—only muscle force	1	[22]
Weightbearing single leg	Inward rotatory stress leg on platform—3 kg	1	[22]
Weightbearing single leg	Outward rotatory stress—only muscle force	1	[22]
Weightbearing single leg	Outward rotatory stress leg on platform—3 kg	1	[22]
Weightbearing single leg	Squatting	1	[22]
Weightbearing single leg	Squatting 70°	1	[21]
Weightbearing valgus stress – 10 kg	Weightbearing varus stress – 10 kg	1	[32]
Weightbearing varus stress – 10 kg	Weightbearing valgus stress – 10 kg	1	[32]

**Table 3:** Maximum and minimum MTPM for inducible displacement examinations

Follow-up, months	Tibial baseplates	Weighted mean MTPM (SD) ^a^	Highest MTPM (SD) ^b^	Examination for maximum	Lowest MTPM (SD) ^b^	Examination for minimum
> 12	Cemented TKA	0.42 (0.20)	0.77 (0.36) at > 120 months	supine vs weightbearing both legs	0.12 (NA) at 60 months	supine vs WBSL
> 12	Cemented UKA	0.32 (0.03)	0.35 (0.15) at 24 months	WBSL vs outward rotatory stress leg on platform—3 kg	0.26 (0.2) at 24 months	WBSL vs inward rotatory stress – only muscle force
> 12	Cementless TKA	0.36 (0.11)	0.49 (0.13) at 24 months	Outward rotatory stress leg on platform—10 Nm vs inward rotatory stress leg on platform—10 Nm	0.18 (0.11) at > 120 months	supine vs WBSL
≤ 12	Cemented TKA	0.35 (0.07)	0.48 (0.21) at3 months	Supine vs WBSL	0.29 (0.12) at 24–48 months	supine vs outward rotatory stress leg on platform—10 Nm
≤ 12	Cemented UKA	0.39 (0.07)	0.47 (0.71) at 12 months	WBSL vs outward rotatory stress leg on platform—3 kg	0.3 (0.09) at 12 months	supine vs WBSL
≤ 12	Cementless TKA	0.45 (0.42)	1.24 (NA) at 1.5 months	Supine vs WBSL	0.05 (0) at 12 months	WBSL vs squatting 70°

a Mean values are sample-size weighted descriptive summaries calculated for illustrative purposes only. They do not constitute pooled meta-analytic estimates and should not be interpreted as such given the substantial heterogeneity in loading protocols, implant types, and follow-up durations across contributing studies.

b Highest and lowest values are drawn from individual studies using distinct loading protocols and follow-up durations and should not be interpreted as the range of a comparable set of measurements.

MTPM = maximum total point migration. WBSL = weightbearing single leg.

### MTPM of multiple weightbearing stress examinations at the same follow-up time point

**Figure 2:**
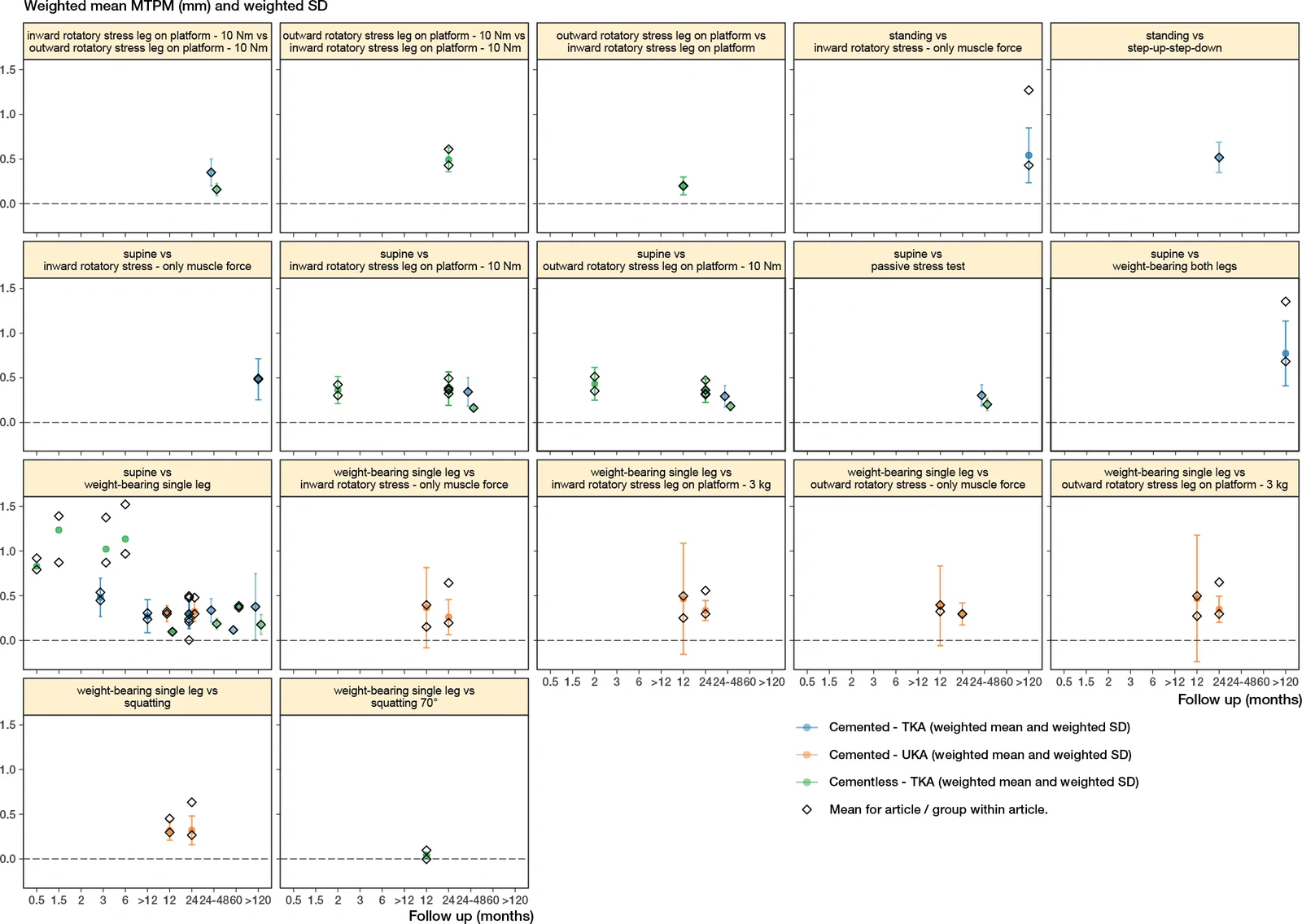
Maximum total point migration (MTPM) for different examinations at different follow-up points for different prosthesis combinations. Mean values and standard devations shown are sample-size weighted descriptive summaries presented ofr illustrative purpose only. They do not represent pooled meta-analytic estimates. Given the heterogeneity of loading protocols, implant types, and follow-up durations across contributing studies, individual data points should be interpreted independently rather than as components of comparable dataset. The figure is intended to provide a visual overview of distribution of reported values and does not support quantitative cross-study comparison.

7 studies conducted multiple inducible displacement examinations at the same follow-up time point, all of which reported MTPM values (Table A5, see Supplementary data). Within individual studies, differences in MTPM between loading conditions at the same time point were generally small, though considerable variability was observed across studies in both the magnitude and direction of these differences. For descriptive orientation only, sample-size weighted means and standard deviations are presented in Figure 2 and Table A6 (see Supplementary data). These summaries do not constitute pooled meta-analytic estimates and should not be interpreted as such, given the substantial heterogeneity in loading protocols, implant types, and follow-up durations across contributing studies.

### Differences and correlations between weightbearing single-leg inducible displacement and same-day supine RSA migration MTPM

**Figure 3:**
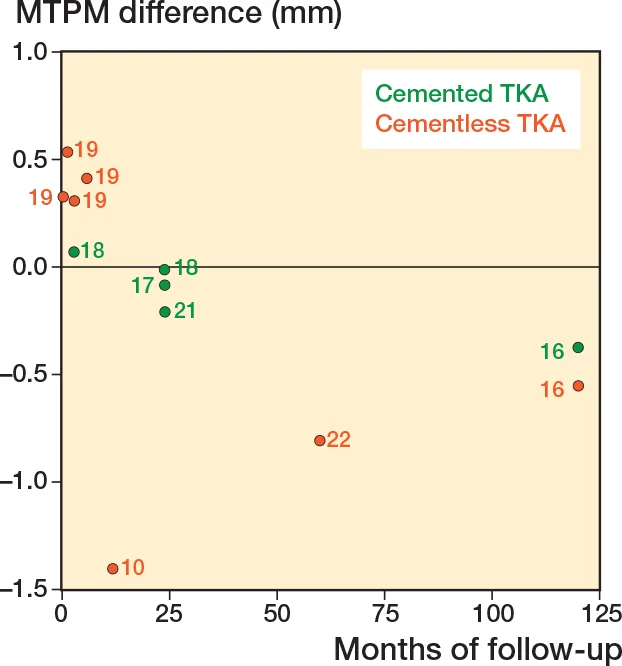
Difference between weightbearing single-leg RSA and supine RSA at same follow-up by follow-up time. Numbers are reference number. MTPM = maximum total point migration, RSA = radiostereometric analysis, TKA = total knee arthroplasty.

8 studies reported both single-leg weightbearing inducible displacement MTPM and same-day supine migration MTPM (Table A7, see Supplementary data), most commonly at 24 months (n = 3), followed by 3 and > 1^20^ months (n = 2 each). Within-study differences are shown in Figure 3 for descriptive orientation only.

### Studies with longitudinal inducible displacement examinations

4 studies reported longitudinal inducible displacement results (Table A8, see Supplementary data), covering 5 tibial baseplates: 1 cemented UKA and 4 TKAs (1 cemented, 3 cementless). 3 compared supine and single-leg weightbearing conditions.

Broberg et al. assessed cementless TKA fixation using MRI, radiographs, and RSA, classifying fixation as normal or fibrous based on MRI findings [27]. Inducible displacement increased progressively in the fibrous group while remaining stable in the normal fixation group (Figure A1, see Supplementary data). In a separate study, Broberg et al. found no significant difference in inducible displacement between knee arthroplasties performed with gap-balancing and measured resection techniques (Figure A2, see Supplementary data) [16].

Bragonzoni et al. reported the only longitudinal UKA data, examining the cemented Duracon UNI prosthesis at 1- and 2-year follow-ups [22]. Patients were grouped by pain status: those with pain (n = 2) and those without (n = 14). In the pain group, inducible displacement increased across nearly all conditions between 1 and 2 years. By 2 years, this group showed the highest displacement in all but one condition (Figure A3, see Supplementary data).

Regnér et al. found that hydroxyapatite-coated cementless TKAs exhibited less inducible micromotion than porous-coated designs, using supine vs inward/outward rotatory stress comparisons at 10 Nm (Figure A4, see Supplementary data) [23].

Only 2 studies, both by Broberg et al., reported both longitudinal supine RSA and inducible displacement measurements [16,27]. Only the fibrous vs normal fixation study included more than 2 follow-up time points.

### Inducible displacement recorded with dynamic RSA

Horsager et al. assessed cemented and cementless UKAs during a step-up/step-down cycle using dynamic RSA (10 fps), finding no significant difference between fixation types. Peak MTPM occurred during the stance phase (0.54 mm, CI 0.44 to 0.65) [19]. Uvehammer et al. used dynamic RSA (3–4 fps) during 8 cm step ascent, reporting MTPM of 0.37–0.52 mm during knee extension from 45° to 15°, increased inducible displacement at 20° of flexion appeared associated with greater longitudinal migration over 2 years [37].

### Relationship between inducible displacement and at-risk implants

Hilding et al. found that continuously migrating unstable cementless tibial baseplates showed greater inducible displacement than stable components, with MTPM differences of approximately 0.1–0.2 mm depending on the examination performed [30]. Horsager et al. reported that the patient with the highest inducible displacement during a step-up/step-down exercise also had the highest continuous migration (1.3 mm MTPM supine RSA, 2–5 years), exhibiting 0.83° inducible micromotion vs a cohort mean of 0.34° (CI 0.23–0.44) [19]. Wilson et al. found the strongest correlation between 1 and 2 year MTPM and inducible displacement for supine vs inward torque (R² = 0.491), with a weaker correlation for supine vs standing (R² = 0.161) [20]. Regner et al. reported that inducible displacement at 12 months (supine vs inward torque) was associated with higher MTPM at 5 years (r = 0.39) [23]. Cheung et al. observed a correlation between 10-year MTPM and inducible displacement during supine vs internal rotation (R² = 0.45) [35]. However, Teeter et al. found no significant difference in inducible displacement between stable and continuously migrating cementless tibial baseplates at 6 months and 1 year (full weightbearing, both legs) [28].

### Association between bone mineral density (BMD) and inducible displacement

Only 1 study examined the relationship between preoperative BMD and inducible displacement. Petersen et al. found no significant correlation between BMD and inducible displacement at 1 year postoperatively (r = 0.20) [29]. However, preoperative BMD was moderately to strongly correlated with longitudinal non-weightbearing migration MTPM at 6 weeks (r = 0.47), 1 year (r = 0.68), and 3 years (r = 0.54) [29].

### Other factors correlating and influencing inducible displacement

#### Inducible displacement differences between cemented and cementless tibial components

Laende et al. reported higher inducible displacement in cemented than cementless components at 10 years (0.2 mm MTPM, IQR 0.2–0.4 vs 0.1 mm, IQR 0.1–0.2). [18] Wilson et al. also observed higher displacement in cemented than cementless Trabecular Metal Monoblock components at 24–48 months (0.34 mm, SD 0.13 vs 0.19 mm, SD 0.06) [20]. Horsager et al. found no significant difference during dynamic RSA step-up/step-down at 4.4 years [19].

#### Radiolucent lines

Horsager et al. found patients with radiolucent lines (RLL) had 0.5° higher medio-lateral tilt during step-up/step-down dynamic RSA than those without (CI 0.18–0.81, n = 5) [19]. Cheung et al. reported higher inducible displacement in 2 patients with RLL (1.35 mm, SD 0.38 vs 0.68 mm, SD 0.36 without RLL) [35]. Given the very small number of patients with RLL, these figures are illustrative only.

### Overall certainty of evidence

As noted in the “Methods” section, formal GRADE assessment was not performed. Across all synthesized findings, the certainty of the evidence is considered low to very low, reflecting the small number of contributing studies for most comparisons, substantial methodological heterogeneity, high risk of bias in several observational studies, and inconsistent reporting of variance measures. All findings should be interpreted as preliminary descriptive observations rather than established clinical evidence.

## Discussion

This systematic review aimed to clarify the relationship between inducible displacement and longitudinal RSA measurements and to identify cut-off values for at-risk implants. This was not feasible due to the limited number of studies, significant heterogeneity in reported outcomes, methodological variation in examination protocols, and insufficient longitudinal data. Consequently, a formal meta-analysis was not performed but, rather, a descriptive evidence synthesis.

Inducible displacement examinations can generally be grouped into 3 categories: (i) single-leg weightbearing, (ii) rotatory stress tests, and (iii) dynamic RSA studies. Single-leg weightbearing was the most frequently used protocol (18 of 23 studies) and requires no specialized equipment beyond standard RSA facilities, making it the most practically feasible option. Rotatory stress tests were used in fewer studies and require additional equipment and patient cooperation. Direct evidence-based comparison between protocols was not possible given the heterogeneity of the literature; however, the frequency of use and logistical accessibility of single-leg weightbearing suggest it as a reasonable candidate for future standardization efforts.

### Factors influencing inducible displacement

2 of 3 studies reported higher inducible displacement in cemented than in cementless designs, though the evidence was limited and heterogeneous. This may reflect the presence of 3 interfaces in cemented implants, prosthesis–cement, cement–bone, and fibrous tissue, vs a single bone–implant interface in cementless designs, with displacement in cemented components reflecting cement elasticity and in cementless components reflecting bone elasticity.

### Timing of inducible displacement examinations

Most studies included in this review performed inducible displacement examinations after the first postoperative year. 1 study reported higher inducible displacement values within the first year, aligning with the known pattern of migration in cementless implants, which often continues up to 1 year before stabilizing [27]. Therefore, conducting inducible displacement assessments both before and after the 1-year follow-up may provide more comprehensive insight into implant stability over time, especially for cementless implants.

### Micromotion limits for osseointegration and inducible displacement

Early in vivo studies suggested a micromotion threshold of approximately 150 µm for optimal osseointegration [38]. However, a 20^21^ systematic review found no clear upper limit for micromotion, instead demonstrating that osseointegration depends on experimental conditions such as loading frequency, rest periods after initial loading, and study duration, as well as implant-specific factors including surface coating, pore size, and material [38]. The 150 µm threshold is not universally applicable and varies according to mechanical and biological conditions. Therefore, the displacement magnitudes reported in these studies do not necessarily preclude long-term implant stability.

### Limitations

We found significant heterogeneity and a relatively low number of inducible displacement studies compared with the migration literature. Inconsistent parameter reporting, including the absence of variability measures in several studies, further limits comparability. Many included studies were conducted before 2000, involving implants that may no longer be in use, RSA standardization and analysis software have since advanced considerably. The late PROSPERO publication represents a potential source of bias. Terminology restrictions may have excluded relevant literature, as studies of implant micromotion or CT-based methods may not explicitly use the terms “inducible displacement” or “radiostereometric analysis.” The combination of methodological heterogeneity, small sample sizes, and inconsistent reporting precluded the definition of clinical cut-off values. All comparative statements should be interpreted as descriptive patterns rather than quantitative effect estimates.

### Challenges in RSA measurement

We could not establish cut-off values separating at-risk from stable implants. Future studies should pair inducible displacement examinations with longitudinal RSA at matched time points, from postoperatively to 5 years, with revision data at 10–20 years. As CT-RSA runs on conventional scanners, it may broaden the use of RSA for assessing implant stability. The optimal loading protocol remains undefined: weightbearing CT is the most physiological but is still rarely available, whereas a controlled varus–valgus load is simpler and more reproducible, because isolating tibial from femoral rotation is technically difficult [37].

### Conclusion

No threshold identifying at-risk implants can currently be derived from the published RSA literature. 2 of 3 studies comparing fixation methods reported higher displacement in cemented than cementless implants, though based on limited and heterogeneous evidence. Rotatory loading tended to produce higher absolute values where multiple protocols were reported, though cross-study comparison was not possible. Single-leg weightbearing was the most feasible and frequently reported protocol.

*In perspective*, current evidence does not support its formal designation as a universal standard, though it remains a reasonable candidate pending prospective validation. The optimal CT-RSA loading exercise has yet to be determined.

### Supplementary data

Search strategy, Figures A1–A4, and Tables A1–A8 are available as Supplementary data on the article home page, doi: 10.2340/17453674.2026.46318

## Supplementary Material



## Notes

JHJ-L and MS conceived and designed the review. JHJ-L and JFT independently screened titles, abstracts, and full texts, and EL resolved disagreements. JHJ-L extracted the data and performed the descriptive analyses. JHJ-L and JFT assessed risk of bias. JHJ-L drafted the manuscript, and ETP, SR, MS, and EL contributed to interpretation of the findings. All authors revised the manuscript critically and approved the final version.

Handling co-editors: Bart Pijls and Robin Christensen

*Acta* thanks Stephan Maximilian Röhrl and Matthew G Teeter for help with peer review of this manuscript.
