# Supplementary material for: Inducible displacement measured with radiostereometric analysis in primary knee arthroplasty: a systematic review of clinical studies
Source: Acta Orthop. 2026 Sep 23;97:698–707. doi: 10.2340/17453674.2026.46318 (PMC13601866; doi:10.2340/17453674.2026.46318)
Supplement: Supplementary file 1 [file ActaO-97-46318-s1.pdf]

Supplementary data

Search strategy

Pubmed:
Date of search: 30.04.2025
Search string: Arthroplasty, Replacement, Knee"[MeSH] AND ("Radiostereometric Analysis"[MeSH] OR "radiostereometric analysis" OR Radiostereometric analysis OR "RSA" OR RSA OR "radiostereometric measurements") AND (inducible micromotion OR inducible migration OR inducible displacement)

Web of Science (Web of Knowledge)
Date of search: 30.04.2025
Search string: TS=(arthroplast* OR "knee replacement")
AND TS=("radiostereometric analys*" OR "radiostereometric measurement*" OR RSA)
AND TS=("inducible micromotion" OR "inducible migration" OR "inducible displacement")

Scopus
Date of search: 30.04.2025
Search string: (knee replacement OR knee arthroplasty OR total knee replacement OR total knee arthroplasty OR knee prosthesis OR unicompartmental knee arthroplasty) AND (radiostereometric analysis OR radiostereometric measurements OR RSA OR radiostereometry OR migration analysis OR implant migration) AND (inducible micromotion OR inducible migration OR inducible displacement OR early migration OR micromotion OR implant movement OR migration)

Embase
Date of search: 30.04.2025
Search string: ('knee replacement'/exp OR 'knee arthroplasty'/exp OR 'total knee replacement'/exp OR 'total knee arthroplasty'/exp OR 'knee prosthesis'/exp OR 'unicompartmental knee arthroplasty'/exp OR knee replacement:ti,ab,kw OR knee arthroplasty:ti,ab,kw OR total knee replacement:ti,ab,kw OR total knee arthroplasty:ti,ab,kw OR knee prosthesis:ti,ab,kw OR unicompartmental knee arthroplasty:ti,ab,kw) AND ('radiostereometric analysis'/exp OR 'radiostereometric measurements'/exp OR RSA:ti,ab,kw OR radiostereometry:ti,ab,kw OR migration analysis:ti,ab,kw OR implant migration:ti,ab,kw) AND (inducible micromotion:ti,ab,kw OR inducible migration:ti,ab,kw OR inducible displacement:ti,ab,kw OR early migration:ti,ab,kw OR micromotion:ti,ab,kw OR implant movement:ti,ab,kw OR migration:ti,ab,kw)

Appendix, Figure 1. A multimodal assessment of cementless tibial baseplate fixation using radiography, radiostereometric analysis, and magnetic resonance imaging[25]

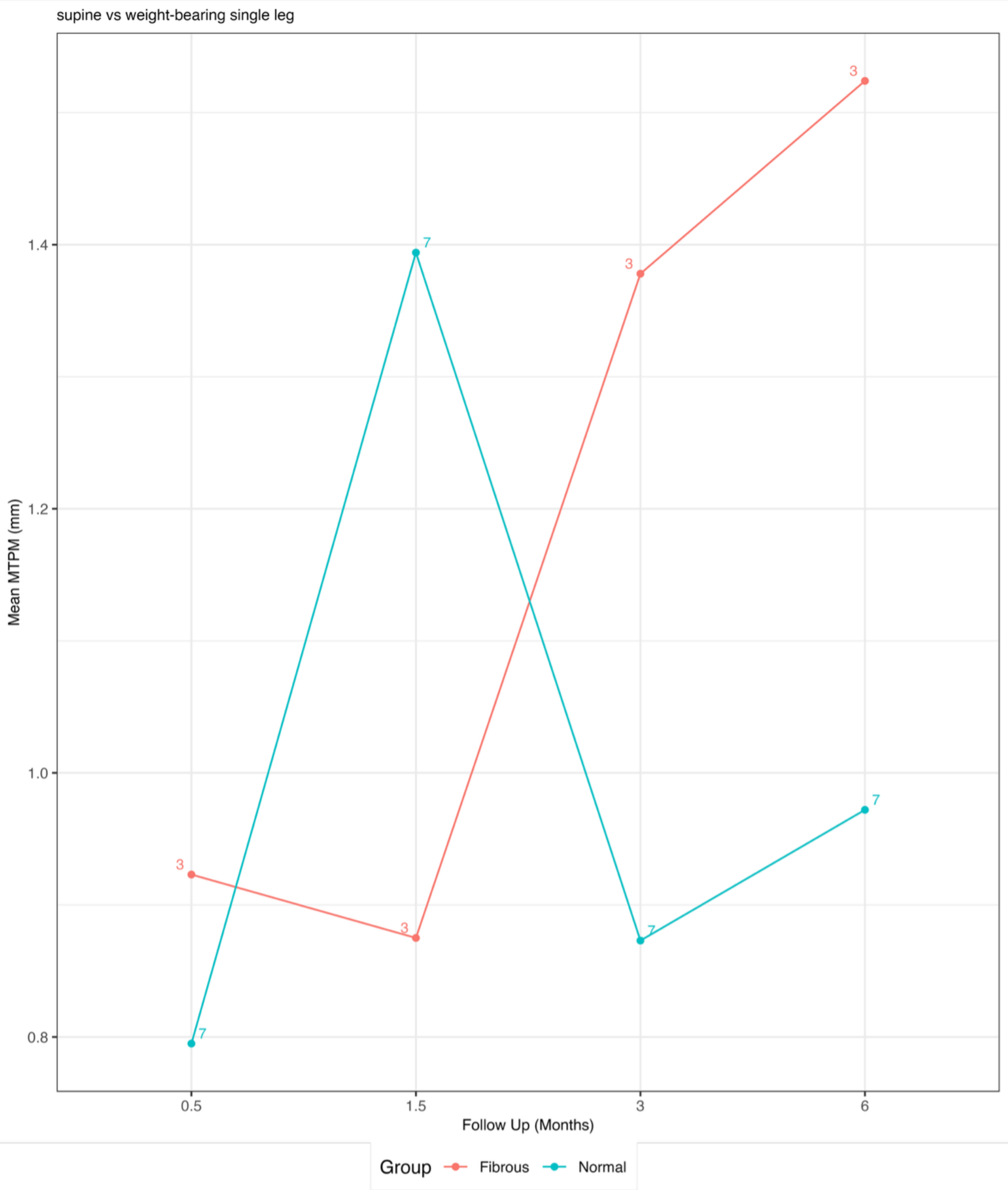

Numbers indicate number of patients.

Appendix, Figure 2. Migration and Inducible Displacement of the Bicruciate-Stabilized Total Knee Arthroplasty: A randomized Controlled Trial of Gap Balancing and Measured Resection Techniques [14]

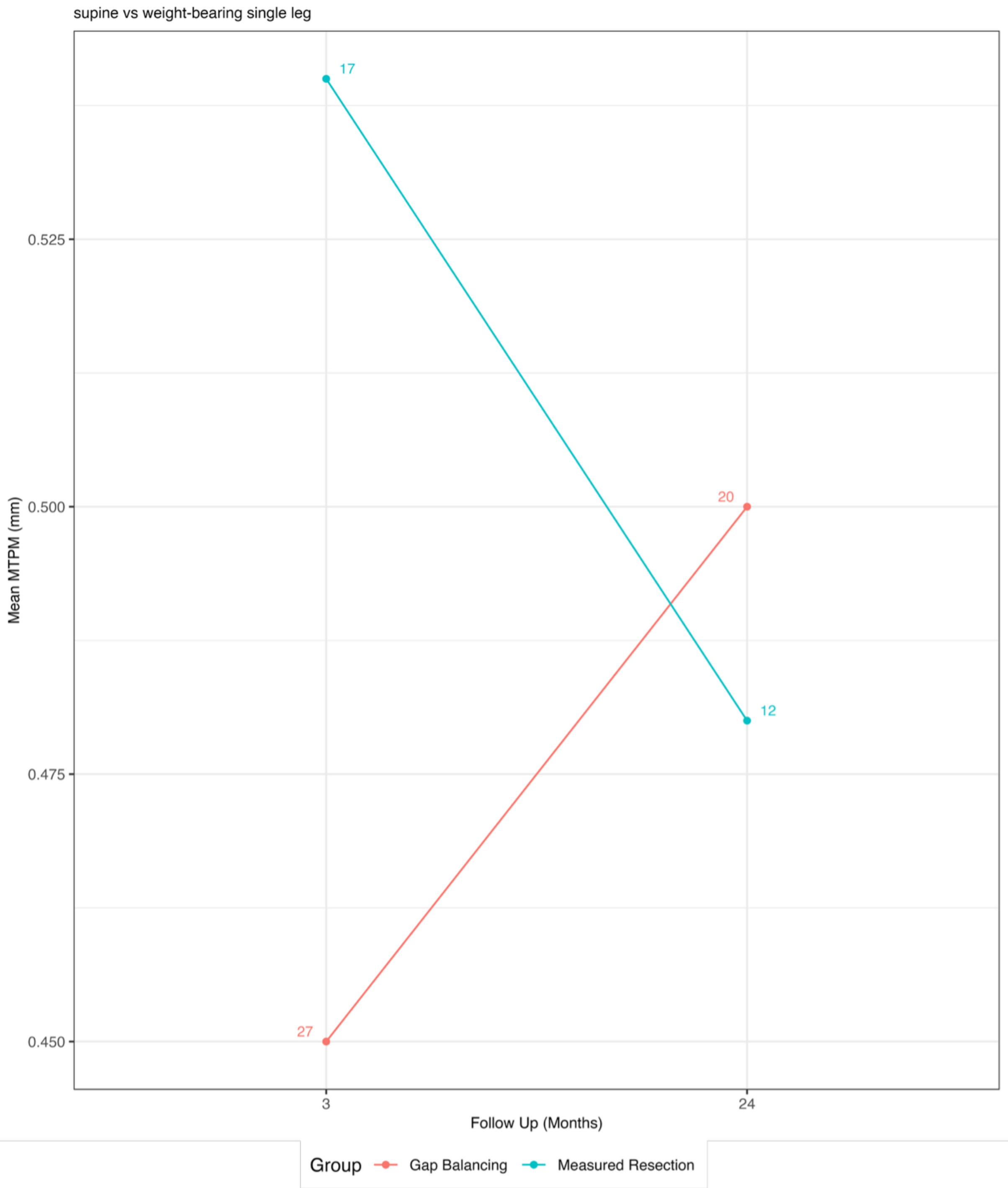

Numbers indicate number of patients.

Appendix, Figure 3. The stress inducible displacement detected through RSA in non-migrating UKR[20]

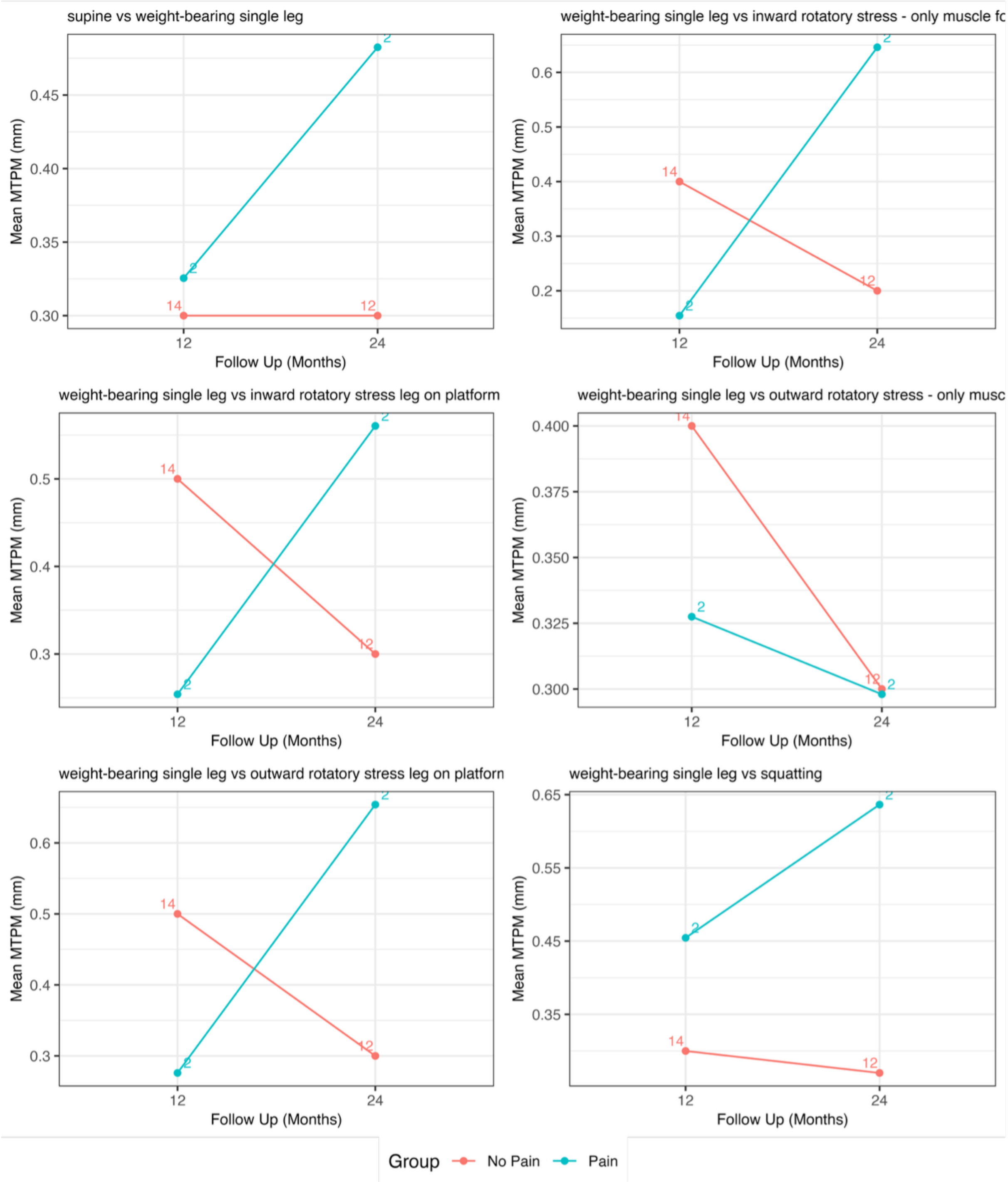

Numbers indicate number of patients.

Appendix, Figure 4. Tibial Component Fixation in Porous- and Hydroxyapatite-Coated Total Knee Arthroplasty - A radiostereometric evaluation of migration and inducible displacement after 5 years[21]

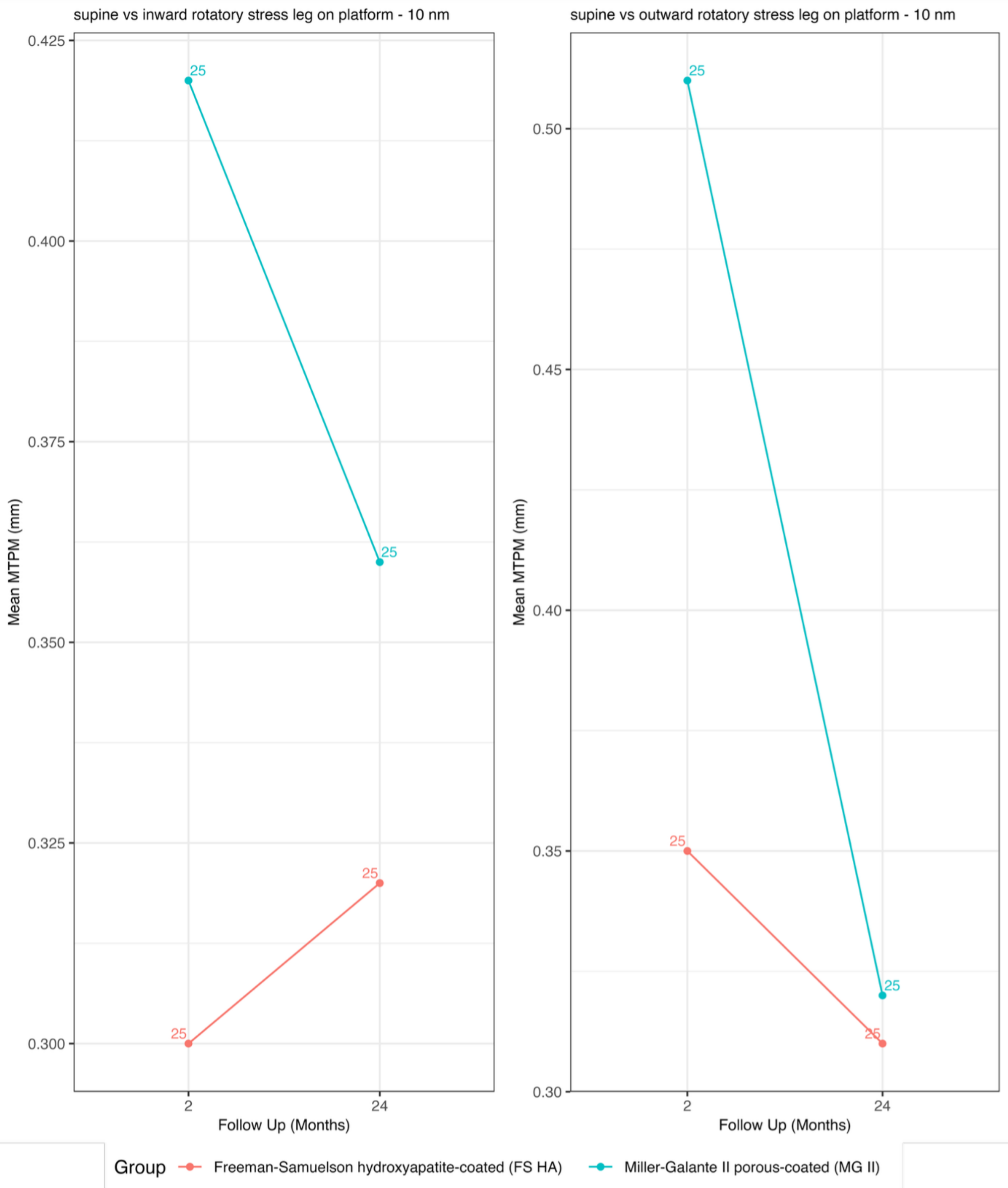

Numbers indicate number of patients.

Appendix Table 1. Studies excluded after full text review

NR	Title	DOI / Link	Author / Year	Exclusion Reason	Details
1	Total knee replacement: intraoperative and postoperative kinematicassessment	Doi: 10.23750/abm.v88i2	Bontempi, M / 2017	Wrong study design	A study investigating kinematics of bones and not of implant movement.
2	New design total knee arthroplasty shows medial pivoting movement underweight-bearing conditions	Doi: 10.1007/s00167-018-5243-5	Bragonzoni, Laura / 2019	No Inducible Micromotion Measured (No IMM)	A kinematic study, no values on inducible displacements are provided.
3	Micromotion of the total knee.	Doi: 10.3109/17453678909149315	Broström, L. A. / 1989	No Inducible Micromotion Measured (No IMM)	Only supine RSA values reported. No inducible displacement examinations are performed.
4	Roentgen stereophotogrammetric analysis: an effective tool to predictimplant survival after an all-poly unicompartmental knee arthroplasty-a 10year follow-up study	Doi: 10.1007/s00167-014-3106-2	Bruni, Danilo / 2015	No Inducible Micromotion Measured (No IMM)	Only supine RSA values reported. No inducible displacement examinations are performed.
5	Accuracy and precision of Volumetric Matching Micromotion Analysis (V3MA) is similar to RSA for tibial component migration in TKA	Doi: 10.1002/jor.25989	de Laat, NN / 2024	No Inducible Micromotion Measured (No IMM)	No Inducible Micromotion Measured (No IMM). No study on humans.
6	Estimation of the migration of tibial components in total kneearthroplasty. A roentgen stereophotogrammetric analysis	PMID:10755430/	Fukuoka, S / 2000	No Inducible Micromotion Measured (No IMM)	Only supine RSA values reported. No inducible displacement examinations are performed.

7	Comparison of micromotion in mobile bearing and posterior stabilized totalknee prostheses: a randomized RSA study of 40 knees followed for 2 years	Doi: 10.1080/00016470510030823	Garling, Eric H / 2005	No Inducible Micromotion Measured (No IMM)	Only supine RSA values reported. No inducible displacement examinations are performed.
8	A Randomised RSA Study of Peri-Apatite™ HA coating of a Knee Prosthesis	Doi: 10.1016/j.knee.2008.01.013	Hansson, U. / 2004	No Inducible Micromotion Measured (No IMM)	Only supine RSA values reported. No inducible displacement examinations are performed.
9	All-polyethylene vs. metal-backed tibial component in total kneearthroplasty-a randomized RSA study comparing early fixation ofhorizontally and completely cemented tibial components: part 2. Completelycemented components: MB not superior to AP components	Doi: 10.1080/17453670510045363	Hyldahl, Hans / 2005	No Inducible Micromotion Measured (No IMM)	Only supine RSA values reported. No inducible displacement examinations are performed.
10	Does metal backing improve fixation of tibial component in unicondylarknee arthroplasty? A randomized radiostereometric analysis	Doi: 10.1054/arth.2001.20257	Hyldahl, H C / 2001	No Inducible Micromotion Measured (No IMM)	Only supine RSA values reported. No inducible displacement examinations are performed.
11	Revision Total Knee Arthroplasty With the Use of Trabecular Metal Cones A Randomized Radiostereometric Analysis With 2 Years of Follow-Up	Doi: 10.1016/j.arth.2012.04.036	Jensen, CL / 2012	No Inducible Micromotion Measured (No IMM)	No inducible displacement values reported. No primary knee arthroplasties.
12	Kinematics of successful knee prostheses during weight-bearing: Three-dimensional movements and positions of screw axes in the Tricon-M and Miller-Galante designs	Doi: 10.1007/BF01552655	Kärrholm, J. / 1994	No Inducible Micromotion Measured (No IMM)	Kinematic values provided no inducible displacement values.

13	Comparable stability of cemented vs press-fit placed stems in revisiontotal knee arthroplasty with mild to moderate bone loss: 6.5-year resultsfrom a randomized controlled trial with radiostereometric analysis	Doi: 10.1016/j.arth.2016.06.003	Kosse, Nienke M / 2017	No Inducible Micromotion Measured (No IMM)	Only supine RSA values reported. No inducible displacement examinations are performed.
14	Major bone transplantation in total knee arthroplasty: a 2- to 9-yearradiostereometric analysis of tibial implant stability	Doi: 10.1016/s0883-5403(99)90117-1	Lindstrand, A / 1999	No Inducible Micromotion Measured (No IMM)	Only supine RSA values reported. No inducible displacement examinations are performed.
15	Micromotion between the half bearings in the interax prosthesis: aroentgen stereophotogrammetric analysis	Doi: 10.1054/arth.2001.25560	Marcacci, M / 2001	No Inducible Micromotion Measured (No IMM)	Only supine RSA values reported. No inducible displacement examinations are performed.
16	IN VIVO KINEMATIC EVALUATION OF A NEW DESIGN TOTAL KNEE ARTHROPLASTY USING DYNAMIC RADIOSTEREOMETRIC ANALYSIS	Doi: 10.1136/jisakos-2020-final-selected-abstracts.7	Marcheggiani-Muccioli, G.M. / 2020	No full text available	Full text was not avaible. Tried access via the Universities library and online resources.
17	Fluoroscopic and radiostereometric analysis of a bicruciate-retaining versus a posterior cruciate-retaining total knee arthroplasty: a randomized controlled trial	Doi: 10.1302/0301-620X.105B1.BJJ-2022-0465.R2	Mills, K. / 2023	No Inducible Micromotion Measured (No IMM)	Only supine RSA values reported. No inducible displacement examinations are performed.
18	A randomized RSA study concentrating especially on continuous migration	Doi: 10.3109/17453674.2016.1166876	Molt, Mats / 2016	No Inducible Micromotion Measured (No IMM)	Only supine RSA values reported. No inducible displacement examinations are performed.

19	Low tibial baseplate migration 1 year after unrestricted kinematically aligned total knee arthroplasty using a medial conforming implant design.	Doi: 10.1007/s00167-022-07171-4	Niesen, Abigail E. / 2023	No Inducible Micromotion Measured (No IMM)	Only supine RSA values reported. No inducible displacement examinations are performed.
20	Scintimetry after total knee arthroplasty: Prospective 2-year study of 18 cases of arthrosis and 15 cases of theumatoid arthritis	Doi: 10.3109/17453679209154814	Nilsson, K.G. / 1992	No Inducible Micromotion Measured (No IMM)	Only supine RSA values reported. No inducible displacement examinations are performed.
21	Evaluation of micromotion in cemented vs uncemented knee arthroplasty in osteoarthrosis and rheumatoid arthritis. Randomized study using roentgen stereophotogrammetric analysis.	Doi: 10.1016/s0883-5403(06)80174-9	Nilsson, K. G. / 1991	No Inducible Micromotion Measured (No IMM)	Only supine RSA values reported. No inducible displacement examinations are performed.
22	CORR insights®: Are there differences in micromotion on radiostereometricanalysis between bicruciate- and cruciate-retaining designs in TKA? Arandomized controlled trial	Doi: 10.1097/CORR.0000000000001173	Nizard, Rémy S / 2020	Wrong study design	A opinion / review for CORR nsights
23	Hydroxyapatite augmentation of the porous coating improves fixation of tibial components. A randomised RSA study in 116 patients.	Doi: 10.1302/0301-620x.80b3.7937	Onsten, I. / 1998	No Inducible Micromotion Measured (No IMM)	Only supine RSA values reported. No inducible displacement examinations are performed.
24	Hydroxyapatite augmentation of the porous coating improves fixation of tibial components	Doi: 10.1302/0301-620X.80B3.7937	Önsten, I. / 1998	No Inducible Micromotion Measured (No IMM)	Only supine RSA values reported. No inducible displacement examinations are performed.
25	Hydroxyapatite augmentation of the porous coating improves fixation of tibial	Doi: 10.1302/0301-620X.80B3.7937	Önsten, I / 1998	Duplicate	Duplicate

	components -: A randomised RSA study in 116 patients				
26	A combined RSA and FE study of the implanted proximal tibia: correlationof the post-operative mechanical environment with implant migration	Doi: 10.1016/j.jbiomech.2003.12.004	Perillo-Marcone, A / 2004	No Inducible Micromotion Measured (No IMM)	Only supine RSA values reported. No inducible displacement examinations are performed.
27	On the correlation between micromotion and histology of the bone-cement interface. Report of three cases of knee arthroplasty followed by roentgen stereophotogrammetric analysis.	Doi: 10.1016/s0883-5403(89)80031-2	Ryd, L. / 1989	No Inducible Micromotion Measured (No IMM)	Only supine RSA values reported. No inducible displacement examinations are performed.
28	No difference in terms of radiostereometric analysis between fixed- andmobile-bearing total knee arthroplasty: a randomized, single-blind,controlled trial	Doi: 10.1007/s00167-016-4138-6	Schotanus, M G M / 2017	No Inducible Micromotion Measured (No IMM)	Only supine RSA values reported. No inducible displacement examinations are performed.
29	Bone morphology in relation to the migration of porous-coated anatomicknee arthroplasties : a roentgen stereophotogrammetric andhistomorphometric study in 23 knees	Doi: 10.1016/s0883-5403(03)00111-6	Tägil, Magnus / 2003	No Inducible Micromotion Measured (No IMM)	Only supine RSA values reported. No inducible displacement examinations are performed.
30	Less polyethylene wear in monobloc compared to modularultra-high-molecular-weight-polyethylene inlays in hybrid total kneearthroplasty: A 5-year randomized radiostereometry study	Doi: 10.1016/j.knee.2021.02.033	Torle, Johan / 2021	No Inducible Micromotion Measured (No IMM)	Only supine RSA values reported. No inducible displacement examinations are performed.
31	Flat vs. concave tibial joint surface in total knee arthroplasty -: Randomized evaluation of 39 cases using radiostereometry	Doi: 10.1080/00016470152846583	Uvehammer, J / 2001	No Inducible Micromotion Measured (No IMM)	No RSA values.

32	Periapatite may not improve micromotion of knee prostheses in rheumatoidarthritis	Doi: 10.1097/01.blo.0000224005.18013.ee	van der Linde, Matthys J A / 2006	No Inducible Micromotion Measured (No IMM)	Only supine RSA values reported. No inducible displacement examinations are performed.
33	Peri-apatite coating decreases uncemented tibial component migration: long-term RSA results of a randomized controlled trial and limitations of short-term results	Doi: 10.1080/17453674.2018.1469223	van Hamersveld, KT / 2018	No Inducible Micromotion Measured (No IMM)	Only supine RSA values reported. No inducible displacement examinations are performed.
34	Acceptable migration of a fully cemented rotating hinge-type knee revision system measured in 20 patients with model-based RSA with a 2-year follow-up.	Doi: 10.2340/17453674.2023.12305	Van Laarhoven, Simon N. / 2023	No Inducible Micromotion Measured (No IMM)	Only supine RSA values reported. No inducible displacement examinations are performed.
35	Computer assisted versus conventional cemented total knee prosthesesalignment accuracy and micromotion of the tibial component	Doi: 10.1007/s00264-008-0642-8	van Strien, Thisbe / 2009	No Inducible Micromotion Measured (No IMM)	Only supine RSA values reported. No inducible displacement examinations are performed.
36	Pre-operative muscle activation patterns during walking are associatedwith TKA tibial implant migration	Doi: 10.1016/j.clinbiomech.2012.06.012	Wilson, David A J / 2012	No Inducible Micromotion Measured (No IMM)	Only supine RSA values reported. No inducible displacement examinations are performed.

Appendix Table 2. Risk of bias (ROB2)

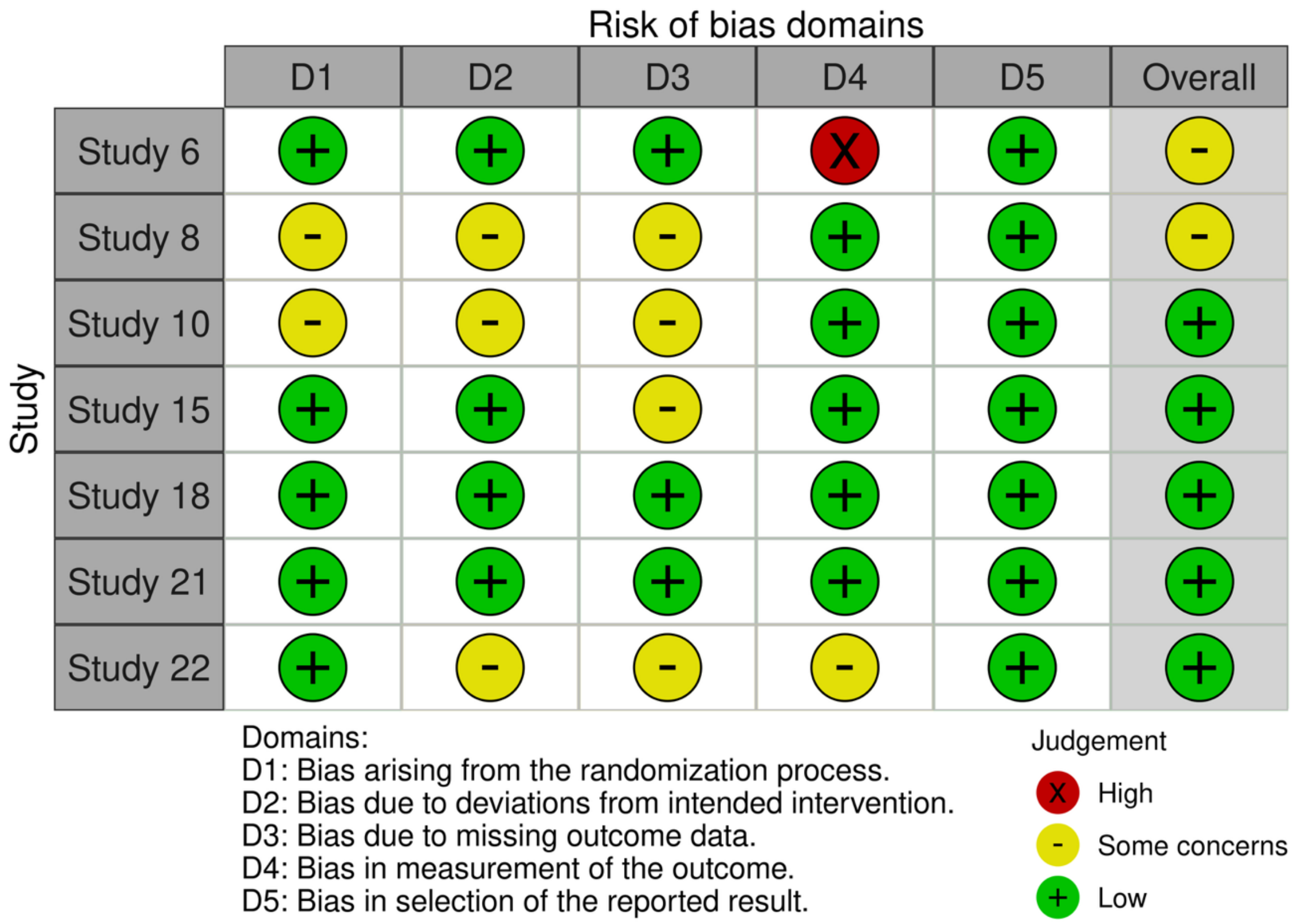

Appendix Table 3. Risk of bias, ROBINS-E

Risk of bias domains

Study	D1	D2	D3	D4	D5	D6	D7	Overall
Study 1	-	+	X	+	-	-	X	X
Study 2	+	+	X	+	-	-	X	-
Study 3	-	+	X	+	X	-	X	X
Study 4	-	+	X	+	X	X	X	X
Study 5	+	+	X	+	X	X	X	X
Study 7	-	+	-	+	X	X	X	X
Study 9	+	-	X	+	X	X	X	X
Study 11	-	+	-	+	-	-	-	-
Study 12	+	+	X	-	+	-	+	-
Study 13	+	+	-	-	-	+	-	-
Study 14	+	+	-	-	-	-	-	+
Study 16	-	+	-	-	+	+	+	+
Study 17	+	+	+	-	-	X	+	-
Study 19	+	+	-	+	-	+	X	-
Study 23	+	+	-	+	-	-	-	+

Domains:
D1: Bias due to confounding.
D2: Bias arising from measurement of the exposure.
D3: Bias in selection of participants into the study (or into the analysis).
D4: Bias due to post-exposure interventions.
D5: Bias due to missing data.
D6: Bias arising from measurement of the outcome.
D7: Bias in selection of the reported result.

Judgement
X High
- Some concerns
+ Low

Appendix Table 4. Raw values extracted from studies

ID	Article	Group / Subgroup	Examination	FU (Mo.)	Fixation	N	MTPM (mm)	Tx (mm)	Ty (mm)	Tz (mm)	Rx (°)	Ry (°)	Rz (°)	TT (mm)	TR (°)
2	Micromotion of Conventionally Cemented All-polyethylene Tibial Components in total knee replacements	—	**outward rotatory stress leg on platform - 10 nm vs inward rotatory stress leg on platform - 10 nm**	24-52	Cemented - TKA	25	N.A.	N.A.	N.A.	N.A.	N.A.	0.47(0.41) [0.00;1.80]	N.A.	N.A.	N.A.
		—	**supine vs weight-bearing single leg**	24-52	Cemented - TKA	25	N.A.	N.A.	N.A.	N.A.	N.A.	N.A.	N.A.	0.30(0.36) [0.00;1.20]	N.A.
6	The stability of three different cementless tibial components	PCA	**outward rotatory stress leg on platform - 10 nm vs inward rotatory stress leg on platform - 10 nm**	24	Cementless - TKA	15	N.A.	N.A.	N.A.	N.A.	N.A.	N.A.	-0.06(0.33)	N.A.	N.A.
		Tricon stem	**outward rotatory stress leg on platform - 10 nm vs inward rotatory stress leg on platform - 10 nm**	24	Cementless - TKA	15	N.A.	N.A.	N.A.	N.A.	N.A.	N.A.	0.25(0.39)	N.A.	N.A.
		Tricon-M	**outward rotatory stress leg on platform - 10 nm vs inward rotatory stress leg on platform - 10 nm**	24	Cementless - TKA	13	N.A.	N.A.	N.A.	N.A.	N.A.	N.A.	0.40(0.40)	N.A.	N.A.
		Stable	**outward rotatory stress leg on platform - 10 nm vs inward rotatory stress leg on platform - 10 nm**	24	Cementless - TKA	28	0.43(0.19)	N.A.	N.A.	N.A.	N.A.	N.A.	N.A.	N.A.	N.A.
		Unstable	**outward rotatory stress leg on platform - 10 nm vs inward rotatory stress leg on platform - 10 nm**	24	Cementless - TKA	15	0.61(0.03)	N.A.	N.A.	N.A.	N.A.	N.A.	N.A.	N.A.	N.A.
		PCA	**supine vs inward rotatory stress leg on platform - 10 nm**	24	Cementless - TKA	15	N.A.	N.A.	N.A.	N.A.	N.A.	N.A.	0.05(0.33)	N.A.	N.A.
		Tricon stem	**supine vs inward rotatory stress leg on platform - 10 nm**	24	Cementless - TKA	15	N.A.	N.A.	N.A.	N.A.	N.A.	N.A.	0.10(0.36)	N.A.	N.A.
		Tricon-M	**supine vs inward rotatory stress leg on platform - 10 nm**	24	Cementless - TKA	13	N.A.	N.A.	N.A.	N.A.	N.A.	N.A.	0.17(0.33)	N.A.	N.A.
		Stable	**supine vs inward rotatory stress leg on platform - 10 nm**	24	Cementless - TKA	28	0.38(0.24)	N.A.	N.A.	N.A.	N.A.	N.A.	N.A.	N.A.	N.A.
		Unstable	**supine vs inward rotatory stress leg on platform - 10 nm**	24	Cementless - TKA	15	0.49(0.27)	N.A.	N.A.	N.A.	N.A.	N.A.	N.A.	N.A.	N.A.
		PCA	**supine vs outward rotatory stress leg on platform - 10 nm**	24	Cementless - TKA	15	N.A.	N.A.	N.A.	N.A.	N.A.	N.A.	0.03(0.26)	N.A.	N.A.
		Tricon stem	**supine vs outward rotatory stress leg on platform - 10 nm**	24	Cementless - TKA	15	N.A.	N.A.	N.A.	N.A.	N.A.	N.A.	-0.19(0.25)	N.A.	N.A.
		Tricon-M	**supine vs outward rotatory stress leg on platform - 10 nm**	24	Cementless - TKA	13	N.A.	N.A.	N.A.	N.A.	N.A.	N.A.	-0.24(0.24)	N.A.	N.A.
		Stable	**supine vs outward rotatory stress leg on platform - 10 nm**	24	Cementless - TKA	28	0.36(0.13)	N.A.	N.A.	N.A.	N.A.	N.A.	N.A.	N.A.	N.A.
		Unstable	**supine vs outward rotatory stress leg on platform - 10 nm**	24	Cementless - TKA	15	0.47(0.19)	N.A.	N.A.	N.A.	N.A.	N.A.	N.A.	N.A.	N.A.
8	Tibial Component Fixation in Porous- and Hydroxyapatite-Coated Total Knee Arthroplasty - A radiostereometric evaluation of migration and inducible displacement after 5 years	Freeman-Samuelson hydroxyapatite-coated (FS HA)	**supine vs inward rotatory stress leg on platform - 10 nm**	2	Cementless - TKA	25	0.30 [0.12;0.51]	N.A.	-0.17 [-0.51;0.12]	N.A.	0.15 [0.00;0.65]	N.A.	N.A.	N.A.	N.A.
		Miller-Galante II porous-coated (MG II)	**supine vs inward rotatory stress leg on platform - 10 nm**	2	Cementless - TKA	25	0.42 [0.18;0.98]	N.A.	-0.22 [-0.56;0.03]	N.A.	0.18 [0.20;0.66]	N.A.	N.A.	N.A.	N.A.

ID	Article	Group / Subgroup	Examination	FU (Mo.)	Fixation	N	MTPM (mm)	Tx (mm)	Ty (mm)	Tz (mm)	Rx (°)	Ry (°)	Rz (°)	TT (mm)	TR (°)
		Freeman-Samuelson hydroxyapatite-coated (FS HA)	**supine vs inward rotatory stress leg on platform - 10 nm**	24	Cementless - TKA	25	0.32 [0.13;0.58]	N.A.	-0.15 [-0.29;0.03]	N.A.	0.10 [0.00;0.27]	N.A.	N.A.	N.A.	N.A.
		Miller-Galante II porous-coated (MG II)	**supine vs inward rotatory stress leg on platform - 10 nm**	24	Cementless - TKA	25	0.36 [0.15;0.75]	N.A.	-0.23 [-0.47;-0.04]	N.A.	0.22 [0.01;0.57]	N.A.	N.A.	N.A.	N.A.
		Freeman-Samuelson hydroxyapatite-coated (FS HA)	**supine vs outward rotatory stress leg on platform - 10 nm**	2	Cementless - TKA	25	0.35 [0.15;0.59]	N.A.	-0.16 [-0.48;0.07]	N.A.	0.18 [0.01;0.62]	N.A.	N.A.	N.A.	N.A.
		Miller-Galante II porous-coated (MG II)	**supine vs outward rotatory stress leg on platform - 10 nm**	2	Cementless - TKA	25	0.51 [0.10;1.10]	N.A.	-0.28 [-0.66;0.04]	N.A.	0.20 [0.02;0.45]	N.A.	N.A.	N.A.	N.A.
		Freeman-Samuelson hydroxyapatite-coated (FS HA)	**supine vs outward rotatory stress leg on platform - 10 nm**	24	Cementless - TKA	25	0.31 [0.15;0.61]	N.A.	-0.16 [-0.42;0.02]	N.A.	0.10 [0.00;0.23]	N.A.	N.A.	N.A.	N.A.
		Miller-Galante II porous-coated (MG II)	**supine vs outward rotatory stress leg on platform - 10 nm**	24	Cementless - TKA	25	0.32 [0.11;0.55]	N.A.	-0.24 [-0.54;-0.06]	N.A.	0.22 [0.03;0.63]	N.A.	N.A.	N.A.	N.A.
9	Inducible displacements of cemented tibial components during weight-bearing and knee extension. Observations during dynamic radiostereometry related to joint positions and 2 years history of migration in 16 TKR	Cemented	**standing vs step-up-step-down**	24	Cemented - TKA	16	0.52(0.17) [0.22;0.82]	N.A.	[-0.21;0.13]	N.A.	-0.07(0.33) [-0.63;0.55]	-0.02(0.43) [-0.72;0.81]	0.12(0.17) [-0.16;0.43]	N.A.	N.A.
10	Mobile vs fixed meniscal bearing in total knee replacement A randomised radiostereometric study	Rotaglide Total Knee System (RTK). Porous coated	**outward rotatory stress leg on platform vs inward rotatory stress log on platform**	12	Cementless - TKA	22	0.20(0.10)	N.A.	N.A.	N.A.	N.A.	N.A.	N.A.	N.A.	N.A.
		Nuffield Total Knee System (NKT). Porous coated	**outward rotatory stress leg on platform vs inward rotatory stress log on platform**	12	Cementless - TKA	19	0.20(0.10)	N.A.	N.A.	N.A.	N.A.	N.A.	N.A.	N.A.	N.A.
		Rotaglide Total Knee System (RTK). Porous coated	**supine vs weight-bearing single leg**	12	Cementless - TKA	22	0.10(0.00)	N.A.	N.A.	N.A.	N.A.	N.A.	N.A.	N.A.	N.A.
		Nuffield Total Knee System (NKT). Porous coated	**supine vs weight-bearing single leg**	12	Cementless - TKA	19	0.10(0.00)	N.A.	N.A.	N.A.	N.A.	N.A.	N.A.	N.A.	N.A.
		Rotaglide Total Knee System (RTK). Porous coated	**weight-bearing single leg vs squatting 70°**	12	Cementless - TKA	22	0.00(0.00)	N.A.	N.A.	N.A.	N.A.	N.A.	N.A.	N.A.	N.A.
		Nuffield Total Knee System (NKT). Porous coated	**weight-bearing single leg vs squatting 70°**	12	Cementless - TKA	19	0.10(0.00)	N.A.	N.A.	N.A.	N.A.	N.A.	N.A.	N.A.	N.A.
11	The stress inducible displacement detected through RSA in non-migrating UKR	No Pain	**supine vs weight-bearing single leg**	12	Cemented - UKA	14	0.30(0.10) [0.14;0.54]	N.A.	N.A.	N.A.	N.A.	N.A.	N.A.	N.A.	N.A.
		Pain	**supine vs weight-bearing single leg**	12	Cemented - UKA	2	0.33 [0.32;0.33]	N.A.	N.A.	N.A.	N.A.	N.A.	N.A.	N.A.	N.A.
		No Pain	**supine vs weight-bearing single leg**	24	Cemented - UKA	12	0.30(0.10) [0.14;0.46]	N.A.	N.A.	N.A.	N.A.	N.A.	N.A.	N.A.	N.A.
		Pain	**supine vs weight-bearing single leg**	24	Cemented - UKA	2	0.48 [0.37;0.60]	N.A.	N.A.	N.A.	N.A.	N.A.	N.A.	N.A.	N.A.
		No Pain	**weight-bearing single leg vs inward rotatory stress - only muscle force**	12	Cemented - UKA	14	0.40(0.50) [0.10;2.00]	N.A.	N.A.	N.A.	N.A.	N.A.	N.A.	N.A.	N.A.
		Pain	**weight-bearing single leg vs inward rotatory stress - only muscle force**	12	Cemented - UKA	2	0.15 [0.10;0.21]	N.A.	N.A.	N.A.	N.A.	N.A.	N.A.	N.A.	N.A.

ID	Article	Group / Subgroup	Examination	FU (Mo.)	Fixation	N	MTPM (mm)	Tx (mm)	Ty (mm)	Tz (mm)	Rx (°)	Ry (°)	Rz (°)	TT (mm)	TR (°)
		No Pain	**weight-bearing single leg vs inward rotatory stress - only muscle force**	24	Cemented - UKA	12	0.20(0.10) [0.08;0.41]	N.A.	N.A.	N.A.	N.A.	N.A.	N.A.	N.A.	N.A.
		Pain	**weight-bearing single leg vs inward rotatory stress - only muscle force**	24	Cemented - UKA	2	0.65 [0.19;1.10]	N.A.	N.A.	N.A.	N.A.	N.A.	N.A.	N.A.	N.A.
		No Pain	**weight-bearing single leg vs inward rotatory stress leg on platform - 3 kg**	12	Cemented - UKA	14	0.50(0.70) [0.18;2.69]	N.A.	N.A.	N.A.	N.A.	N.A.	N.A.	N.A.	N.A.
		Pain	**weight-bearing single leg vs inward rotatory stress leg on platform - 3 kg**	12	Cemented - UKA	2	0.25 [0.22;0.29]	N.A.	N.A.	N.A.	N.A.	N.A.	N.A.	N.A.	N.A.
		No Pain	**weight-bearing single leg vs inward rotatory stress leg on platform - 3 kg**	24	Cemented - UKA	12	0.30(0.10) [0.19;0.59]	N.A.	N.A.	N.A.	N.A.	N.A.	N.A.	N.A.	N.A.
		Pain	**weight-bearing single leg vs inward rotatory stress leg on platform - 3 kg**	24	Cemented - UKA	2	0.56 [0.45;0.67]	N.A.	N.A.	N.A.	N.A.	N.A.	N.A.	N.A.	N.A.
		No Pain	**weight-bearing single leg vs outward rotatory stress - only muscle force**	12	Cemented - UKA	14	0.40(0.50) [0.14;1.88]	N.A.	N.A.	N.A.	N.A.	N.A.	N.A.	N.A.	N.A.
		Pain	**weight-bearing single leg vs outward rotatory stress - only muscle force**	12	Cemented - UKA	2	0.33 [0.29;0.36]	N.A.	N.A.	N.A.	N.A.	N.A.	N.A.	N.A.	N.A.
		No Pain	**weight-bearing single leg vs outward rotatory stress - only muscle force**	24	Cemented - UKA	12	0.30(0.10) [0.15;0.47]	N.A.	N.A.	N.A.	N.A.	N.A.	N.A.	N.A.	N.A.
		Pain	**weight-bearing single leg vs outward rotatory stress - only muscle force**	24	Cemented - UKA	2	0.30 [0.14;0.45]	N.A.	N.A.	N.A.	N.A.	N.A.	N.A.	N.A.	N.A.
		No Pain	**weight-bearing single leg vs outward rotatory stress leg on platform - 3 kg**	12	Cemented - UKA	14	0.50(0.80) [0.17;3.17]	N.A.	N.A.	N.A.	N.A.	N.A.	N.A.	N.A.	N.A.
		Pain	**weight-bearing single leg vs outward rotatory stress leg on platform - 3 kg**	12	Cemented - UKA	2	0.28 [0.24;0.31]	N.A.	N.A.	N.A.	N.A.	N.A.	N.A.	N.A.	N.A.
		No Pain	**weight-bearing single leg vs outward rotatory stress leg on platform - 3 kg**	24	Cemented - UKA	12	0.30(0.10) [0.12;0.45]	N.A.	N.A.	N.A.	N.A.	N.A.	N.A.	N.A.	N.A.
		Pain	**weight-bearing single leg vs outward rotatory stress leg on platform - 3 kg**	24	Cemented - UKA	2	0.65 [0.40;0.91]	N.A.	N.A.	N.A.	N.A.	N.A.	N.A.	N.A.	N.A.
		No Pain	**weight-bearing single leg vs squatting**	12	Cemented - UKA	14	0.30(0.10) [0.13;0.65]	N.A.	N.A.	N.A.	N.A.	N.A.	N.A.	N.A.	N.A.
		Pain	**weight-bearing single leg vs squatting**	12	Cemented - UKA	2	0.45 [0.36;0.55]	N.A.	N.A.	N.A.	N.A.	N.A.	N.A.	N.A.	N.A.
		No Pain	**weight-bearing single leg vs squatting**	24	Cemented - UKA	12	0.27(0.06) [0.19;0.39]	N.A.	N.A.	N.A.	N.A.	N.A.	N.A.	N.A.	N.A.
		Pain	**weight-bearing single leg vs squatting**	24	Cemented - UKA	2	0.64 [0.18;1.09]	N.A.	N.A.	N.A.	N.A.	N.A.	N.A.	N.A.	N.A.
12	Inducible Displacement of a Trabecular Metal Tibial Monoblock Component	Cementless	**inward rotatory stress leg on platform - 10 nm vs outward rotatory stress leg on platform - 10 nm**	24-48	Cementless - TKA	14	0.16(0.07)	N.A.	N.A.	N.A.	N.A.	N.A.	N.A.	N.A.	N.A.
		Cemented	**inward rotatory stress leg on platform - 10 nm vs outward rotatory stress leg on platform - 10 nm**	24-48	Cemented - TKA	11	0.35(0.15)	N.A.	N.A.	N.A.	N.A.	N.A.	N.A.	N.A.	N.A.
		Cementless	**supine vs inward rotatory stress leg on platform - 10 nm**	24-48	Cementless - TKA	14	0.16(0.03)	N.A.	N.A.	N.A.	N.A.	N.A.	N.A.	N.A.	N.A.
		Cemented	**supine vs inward rotatory stress leg on platform - 10 nm**	24-48	Cemented - TKA	11	0.34(0.16)	N.A.	N.A.	N.A.	N.A.	N.A.	N.A.	N.A.	N.A.
		Cementless	**supine vs outward rotatory stress leg on platform - 10 nm**	24-48	Cementless - TKA	14	0.18(0.05)	N.A.	N.A.	N.A.	N.A.	N.A.	N.A.	N.A.	N.A.
		Cemented	**supine vs outward rotatory stress leg on platform - 10 nm**	24-48	Cemented - TKA	11	0.29(0.12)	N.A.	N.A.	N.A.	N.A.	N.A.	N.A.	N.A.	N.A.

ID	Article	Group / Subgroup	Examination	FU (Mo.)	Fixation	N	MTPM (mm)	Tx (mm)	Ty (mm)	Tz (mm)	Rx (°)	Ry (°)	Rz (°)	TT (mm)	TR (°)
		Cementless	**supine vs passive stress test**	24-48	Cementless - TKA	14	0.20(0.07)	N.A.	N.A.	N.A.	N.A.	N.A.	N.A.	N.A.	N.A.
		Cemented	**supine vs passive stress test**	24-48	Cemented - TKA	11	0.30(0.12)	N.A.	N.A.	N.A.	N.A.	N.A.	N.A.	N.A.	N.A.
		Cementless	**supine vs weight-bearing single leg**	24-48	Cementless - TKA	14	0.19(0.06)	N.A.	N.A.	N.A.	N.A.	N.A.	N.A.	N.A.	N.A.
		Cemented	**supine vs weight-bearing single leg**	24-48	Cemented - TKA	11	0.34(0.13)	N.A.	N.A.	N.A.	N.A.	N.A.	N.A.	N.A.	N.A.
14	Inducible displacement of cemented tibial components ten years after total knee arthroplasty	Stable	**standing vs inward rotatory stress - only muscle force**	>120	Cemented - TKA	13	0.43(0.23)	N.A.	N.A.	N.A.	N.A.	N.A.	N.A.	N.A.	N.A.
		Radiolucent	**standing vs inward rotatory stress - only muscle force**	>120	Cemented - TKA	2	1.27(0.81)	N.A.	N.A.	N.A.	N.A.	N.A.	N.A.	N.A.	N.A.
		Stable	**supine vs inward rotatory stress - only muscle force**	>120	Cemented - TKA	13	0.48(0.26)	N.A.	N.A.	N.A.	N.A.	N.A.	N.A.	N.A.	N.A.
		Radiolucent	**supine vs inward rotatory stress - only muscle force**	>120	Cemented - TKA	2	0.49(0.04)	N.A.	N.A.	N.A.	N.A.	N.A.	N.A.	N.A.	N.A.
		Stable	**supine vs weight-bearing both legs**	>120	Cemented - TKA	13	0.68(0.36)	N.A.	N.A.	N.A.	N.A.	N.A.	N.A.	N.A.	N.A.
		Radiolucent	**supine vs weight-bearing both legs**	>120	Cemented - TKA	2	1.35(0.38)	N.A.	N.A.	N.A.	N.A.	N.A.	N.A.	N.A.	N.A.
15	A randomized controlled trial of tibial component migration with kinematic alignment using patient-specific instrumentation versus mechnical alignment using computer-assisted surgery in total knee arthroplasty	KA+PSI	**supine vs weight-bearing single leg**	>12	Cemented - TKA	47	0.31(0.25)	N.A.	N.A.	N.A.	N.A.	N.A.	N.A.	N.A.	N.A.
		MA+CAS	**supine vs weight-bearing single leg**	>12	Cemented - TKA	47	0.24(0.12)	N.A.	N.A.	N.A.	N.A.	N.A.	N.A.	N.A.	N.A.
16	Predictive value of short-term migation in determning long-term stable fixation in cemented and cementless total knee arthroplasties	Cemented	**supine vs weight-bearing single leg**	>120	Cemented - TKA	57	0.38(0.37)	N.A.	N.A.	N.A.	N.A.	N.A.	N.A.	N.A.	N.A.
		Cementless	**supine vs weight-bearing single leg**	>120	Cementless - TKA	21	0.18(0.11)	N.A.	N.A.	N.A.	N.A.	N.A.	N.A.	N.A.	N.A.
17	Tibial component migration after total knee arthroplasty with high-viscocity bone cement	High-Viscocity	**supine vs weight-bearing single leg**	24	Cemented - TKA	23	0.30(0.12)	N.A.	N.A.	N.A.	N.A.	N.A.	N.A.	N.A.	N.A.
		Medium-Viscocity	**supine vs weight-bearing single leg**	24	Cemented - TKA	23	0.24(0.12)	N.A.	N.A.	N.A.	N.A.	N.A.	N.A.	N.A.	N.A.
18	Migration and Inducible Displacement of the Bicruciate-Stabilized Total Knee Arthroplasty: A randomized Controlled Trial of Gap Balancing and Measured Resection Techniques	Gap Balancing	**supine vs weight-bearing single leg**	3	Cemented - TKA	27	0.45 [0.18;0.97]	N.A.	N.A.	N.A.	N.A.	N.A.	N.A.	N.A.	N.A.
		Measured Resection	**supine vs weight-bearing single leg**	3	Cemented - TKA	17	0.54 [0.20;1.07]	N.A.	N.A.	N.A.	N.A.	N.A.	N.A.	N.A.	N.A.
		Gap Balancing	**supine vs weight-bearing single leg**	24	Cemented - TKA	20	0.50 [0.09;1.22]	N.A.	N.A.	N.A.	N.A.	N.A.	N.A.	N.A.	N.A.
		Measured Resection	**supine vs weight-bearing single leg**	24	Cemented - TKA	12	0.48 [0.12;0.89]	N.A.	N.A.	N.A.	N.A.	N.A.	N.A.	N.A.	N.A.
19	A multimodal assessment of cementless tibial baseplate fixation using radiography, radiostereometric analysis, and magnetic resonance imaging	Normal	**supine vs weight-bearing single leg**	0.5	Cementless - TKA	7	0.80	N.A.	N.A.	N.A.	N.A.	N.A.	N.A.	N.A.	N.A.
		Fibrous	**supine vs weight-bearing single leg**	0.5	Cementless - TKA	3	0.92	N.A.	N.A.	N.A.	N.A.	N.A.	N.A.	N.A.	N.A.
		Normal	**supine vs weight-bearing single leg**	1.5	Cementless - TKA	7	1.39	N.A.	N.A.	N.A.	N.A.	N.A.	N.A.	N.A.	N.A.
		Fibrous	**supine vs weight-bearing single leg**	1.5	Cementless - TKA	3	0.88	N.A.	N.A.	N.A.	N.A.	N.A.	N.A.	N.A.	N.A.
		Normal	**supine vs weight-bearing single leg**	3	Cementless - TKA	7	0.87	N.A.	N.A.	N.A.	N.A.	N.A.	N.A.	N.A.	N.A.

ID	Article	Group / Subgroup	Examination	FU (Mo.)	Fixation	N	MTPM (mm)	Tx (mm)	Ty (mm)	Tz (mm)	Rx (°)	Ry (°)	Rz (°)	TT (mm)	TR (°)
		Fibrous	**supine vs weight-bearing single leg**	3	Cementless - TKA	3	1.38	N.A.	N.A.	N.A.	N.A.	N.A.	N.A.	N.A.	N.A.
		Normal	**supine vs weight-bearing single leg**	6	Cementless - TKA	7	0.97	N.A.	N.A.	N.A.	N.A.	N.A.	N.A.	N.A.	N.A.
		Fibrous	**supine vs weight-bearing single leg**	6	Cementless - TKA	3	1.52	N.A.	N.A.	N.A.	N.A.	N.A.	N.A.	N.A.	N.A.
21	Randomized controlled trial comparing traditional versus enhanced-fixation designs of a novel cemented total knee arthroplasty tibial component	Std-TKA	**supine vs weight-bearing single leg**	24	Cemented - TKA	13	0.21(0.15)	N.A.	0.00(0.03)	N.A.	N.A.	N.A.	N.A.	N.A.	N.A.
		EF-TKA	**supine vs weight-bearing single leg**	24	Cemented - TKA	13	0.01(0.04)	N.A.	-0.01(0.04)	N.A.	N.A.	N.A.	N.A.	N.A.	N.A.
22	5-year migration and inducible displacement of the uncemented LCS and ATTUNE rotating platform knee systems: a secondary report of a randomized controlled RSA trial	Attune	**supine vs weight-bearing single leg**	60	Cementless - TKA	24	0.37	-0.04	-0.05	-0.01	-0.01	0.20	0.04	N.A.	N.A.
		LCS	**supine vs weight-bearing single leg**	60	Cementless - TKA	24	0.39	-0.02	-0.04	-0.01	0.03	0.05	0.05	N.A.	N.A.
23	Inducible displacement of cementless total knee arthroplasty components with conventional and weight-bearing CT-based radiostereometric analysis	—	**supine vs weight-bearing single leg**	60	Cemented - TKA	17	0.12	0.01	0.01	0.01	0.00	0.01	-0.01	0.06	0.09

Values presented as Mean(SD) [Min;Max] where available, as reported by the original authors. N.A. = not reported. Ty_Max entries (Study 8, Regner et al.) represent Ty measurements reported under a different label in the source publication and are presented in the Ty column. Max_Ty and Min_Ty entries (Study 9, Uvehammer et al.) represent the cohort mean of maximum and minimum Ty displacement across the motion cycle; presented as [Min_Ty mean; Max_Ty mean]. No cross-study aggregation was performed; all values are drawn directly from individual studies. MTPM = Maximum Total Point Motion (mm). Tx/Ty/Tz = translations (mm). Rx/Ry/Rz = rotations (degrees). TT = Total Translation (mm). TR = Total Rotation (degrees). N = number of participants. Group reflects treatment arms or patient subgroups as defined in the original study; for single-arm studies the label reflects the prosthesis or cohort.

Appendix Table *5* Articles that report multiple inducible examinations at the same follow-up

Article	Follow up (months)	Inducible exami-nations	Inducible examinations
Inducible Displacement of a Trabecular Metal Tibial Monoblock Component [18]	24-48	5	• supine vs weight-bearing single leg<br>• supine vs inward rotatory stress leg on platform - 10 nm<br>• supine vs outward rotatory stress leg on platform - 10 nm<br>• supine vs passive stress test<br>• inward rotatory stress leg on platform - 10 Nm vs outward rotatory stress leg on platform - 10 Nm
Inducible displacement of cemented tibial components ten years after total knee arthroplasty[33]	>120	3	• supine vs weight-bearing both legs<br>• supine vs inward rotatory stress - only muscle force<br>• standing vs inward rotatory stress - only muscle force
Mobile vs fixed meniscal bearing in total knee replacement A randomised radiostereometric study[19]	12	3	• supine vs weight-bearing single leg<br>• outward rotatory stress leg on platform vs inward rotatory stress log on platform<br>• weight-bearing single leg vs squatting 70°
The stability of three different cementless tibial components [28]	24	3	• supine vs outward rotatory stress leg on platform - 10 Nm<br>• supine vs inward rotatory stress leg on platform - 10 Nm<br>• outward rotatory stress leg on platform - 10 Nm vs inward rotatory stress leg on platform - 10 nNm
The stress inducible displacement detected through RSA in non-migrating UKR[20]	12	6	• supine vs weight-bearing single leg<br>• weight-bearing single leg vs inward rotatory stress leg on platform - 3 kg<br>• weight-bearing single leg vs inward rotatory stress - only muscle force<br>• weight-bearing single leg vs outward rotatory stress leg on platform - 3 kg<br>• weight-bearing single leg vs outward rotatory stress - only muscle force<br>• weight-bearing single leg vs squatting
	24	6	• supine vs weight-bearing single leg<br>• weight-bearing single leg vs inward rotatory stress leg on platform - 3 kg<br>• weight-bearing single leg vs inward rotatory stress - only muscle force<br>• weight-bearing single leg vs outward rotatory stress leg on platform - 3 kg<br>• weight-bearing single leg vs outward rotatory stress - only muscle force<br>• weight-bearing single leg vs squatting
Tibial Component Fixation in Porous- and Hydroxyapatite-Coated Total Knee Arthroplasty - A radiostereometric evaluation of migration and inducible displacement after 5 years[21]	2	2	• supine vs inward rotatory stress leg on platform - 10 Nm<br>• supine vs outward rotatory stress leg on platform - 10 Nm
	24	2	• supine vs inward rotatory stress leg on platform - 10 Nm<br>• supine vs outward rotatory stress leg on platform - 10 Nm

Appendix Table 6. MTPM for different examinations at different follow-up points for different prosthesis combinations [1]

		TKA - Cementless					TKA - Cemented					UKA - Cemented				
**Examinations**	**Follow up**	**Mean**	**SD**	**Min**	**Max**	**N**	**Mean**	**SD**	**Min**	**Max**	**N**	**Mean**	**SD**	**Min**	**Max**	**N**
inward rotatory stress leg on platform - 10 nm vs outward rotatory stress leg on platform - 10 nm	24-48	0.16	0.07	0.16	0.16	14	0.35	0.15	0.35	0.35	11					
outward rotatory stress leg on platform - 10 nm vs inward rotatory stress leg on platform - 10 nm	24	0.49	0.13	0.43	0.61	43										
outward rotatory stress leg on platform vs inward rotatory stress log on platform	12	0.20	0.10	0.20	0.20	41										
standing vs inward rotatory stress - only muscle force	>120						0.54	0.31	0.43	1.27	15					
standing vs step-up-step-down	24						0.52	0.17	0.22	0.82	16					
supine vs inward rotatory stress - only muscle force	>120						0.48	0.23	0.48	0.49	15					
supine vs inward rotatory stress leg on platform - 10 nm	24-48	0.16	0.03	0.16	0.16	14	0.34	0.16	0.34	0.34	11					
	24	0.38	0.19	0.13	0.75	93										
	2	0.36	0.15	0.12	0.98	50										
supine vs outward rotatory stress leg on platform - 10 nm	24-48	0.18	0.05	0.18	0.18	14	0.29	0.12	0.29	0.29	11					
	24	0.35	0.13	0.11	0.61	93										

		TKA - Cementless					TKA - Cemented					UKA - Cemented				
**Examinations**	**Follow up**	**Mean**	**SD**	**Min**	**Max**	**N**	**Mean**	**SD**	**Min**	**Max**	**N**	**Mean**	**SD**	**Min**	**Max**	**N**
	2	0.43	0.18	0.10	1.10	50										
supine vs passive stress test	24-48	0.20	0.07	0.20	0.20	14	0.30	0.12	0.30	0.30	11					
supine vs weight-bearing both legs	>120						0.77	0.36	0.68	1.35	15					
supine vs weight-bearing single leg	>120	0.18	0.11	0.18	0.18	21	0.38	0.37	0.38	0.38	57					
	60	0.38		0.37	0.39	48	0.12		0.12	0.12	17					
	24-48	0.19	0.06	0.19	0.19	14	0.34	0.13	0.34	0.34	11					
	24						0.30	0.16	0.01	1.22	104	0.33	0.11	0.14	0.60	14
	12	0.10	0.00	0.10	0.10	41						0.30	0.09	0.14	0.54	16
	>12						0.28	0.18	0.24	0.31	94					
	6	1.14		0.97	1.52	10										
	3	1.02		0.87	1.38	10	0.48	0.21	0.18	1.07	44					
	1.5	1.24		0.88	1.39	10										
	0.5	0.83		0.80	0.92	10										
	24											0.26	0.20	0.08	1.10	14

		TKA - Cementless					TKA - Cemented					UKA - Cemented				
**Examinations**	**Follow up**	**Mean**	**SD**	**Min**	**Max**	**N**	**Mean**	**SD**	**Min**	**Max**	**N**	**Mean**	**SD**	**Min**	**Max**	**N**
weight-bearing single leg vs inward rotatory stress - only muscle force	12											0.37	0.45	0.10	2.00	16
weight-bearing single leg vs inward rotatory stress leg on platform - 3 kg	24											0.34	0.11	0.19	0.67	14
	12											0.47	0.62	0.18	2.69	16
weight-bearing single leg vs outward rotatory stress - only muscle force	24											0.30	0.12	0.14	0.47	14
	12											0.39	0.45	0.14	1.88	16
weight-bearing single leg vs outward rotatory stress leg on platform - 3 kg	24											0.35	0.15	0.12	0.91	14
	12											0.47	0.71	0.17	3.17	16
weight-bearing single leg vs squatting	24											0.32	0.16	0.18	1.09	14
	12											0.32	0.11	0.13	0.65	16
weight-bearing single leg vs squatting 70°	12	0.05	0.00	0.00	0.10	41										

[1] Note: Weighted means and weighted standard deviations presented in this table are sample-size weighted descriptive summaries calculated for illustrative purposes only. They do not constitute pooled meta-analytic estimates and must not be interpreted as such. The substantial heterogeneity across contributing studies in implant design, patient population, surgical technique, and loading protocol means these values carry no inferential weight. Cells with small N values (particularly N = 1 or N = 2) should be interpreted with particular caution, as weighted summaries from one or two studies are not meaningfully distinguishable from the individual study values themselves. Minimum and maximum values derive from individual studies and reflect the observed range across heterogeneous measurements rather than the precision of a comparable set of observations. SD values were extracted directly from articles or estimated from reported ranges using the method of Wan et al. 2014 where not available.

Appendix Table 7. Articles with weight-bearing single leg and supine RSA examinations reporting MTPM values

Article	Follow up	Supine exam	Inducible examination
Migration and Inducible Displacement of the Bicruciate-Stabilized Total Knee Arthroplasty: A randomized Controlled Trial of Gap Balancing and Measured Resection Techniques[14]	3	• supine vs supine	• supine vs weight-bearing single leg
	24	• supine vs supine	• supine vs weight-bearing single leg
Mobile vs fixed meniscal bearing in total knee replacement A randomised radiostereometric study[19]	12	• supine vs supine	• supine vs weight-bearing single leg<br>• outward rotatory stress leg on platform vs inward rotatory stress log on platform<br>• weight-bearing single leg vs squatting 70°
Predictive value of short-term migation in determning long-term stable fixation in cemented and cementless total knee arthroplasties[16]	>120	• supine vs supine	• supine vs weight-bearing single leg
5-year migration and inducible displacement of the uncemented LCS and ATTUNE rotating platform knee systems: a secondary report of a randomized controlled RSA trial[22]	60	• supine vs supine	• supine vs weight-bearing single leg
Inducible displacement of cemented tibial components ten years after total knee arthroplasty[33]	>120	• supine vs supine	• supine vs weight-bearing both legs<br>• supine vs inward rotatory stress - only muscle force<br>• standing vs inward rotatory stress - only muscle force
A multimodal assessment of cementless tibial baseplate fixation using radiography, radiostereometric analysis, and magnetic resonance imaging[25]	0.5	• supine vs supine	• supine vs weight-bearing single leg
	1.5	• supine vs supine	• supine vs weight-bearing single leg

Article	Follow up	Supine exam	Inducible examination
	3	• supine vs supine	• supine vs weight-bearing single leg
	6	• supine vs supine	• supine vs weight-bearing single leg
Randomized controlled trial comparing traditional versus enhanced-fixation designs of a novel cemented total knee arthroplasty tibial component[24]	24	• supine vs supine	• supine vs weight-bearing single leg
Tibial component migration after total knee arthroplasty with high-viscocity bone cement[15]	24	• supine vs supine	• supine vs weight-bearing single leg

Appendix Table 8. List of articles reporting on supine vs weight-bearing single leg

Article	Follow up (Months)	Reference	Migration
Migration and Inducible Displacement of the Bicruciate-Stabilized Total Knee Arthroplasty: A randomized Controlled Trial of Gap Balancing and Measured Resection Techniques[14]	3	supine	weight-bearing single leg
	24	supine	weight-bearing single leg
Tibial Component Fixation in Porous- and Hydroxyapatite-Coated Total Knee Arthroplasty - A radiostereometric evaluation of migration and inducible displacement after 5 years[21]	2	supine	inward rotatory stress leg on platform - 10 Nm
	2	supine	outward rotatory stress leg on platform - 10 Nm
	24	supine	inward rotatory stress leg on platform - 10 Nm
	24	supine	outward rotatory stress leg on platform - 10 Nm
The stress inducible displacement detected through RSA in non-migrating UKR[20]	12	supine	weight-bearing single leg
	12	weight-bearing single leg	inward rotatory stress leg on platform - 3 kg
	12	weight-bearing single leg	inward rotatory stress - only muscle force
	12	weight-bearing single leg	outward rotatory stress leg on platform - 3 kg
	12	weight-bearing single leg	outward rotatory stress - only muscle force
	12	weight-bearing single leg	squatting

Article	Follow up (Months)	Reference	Migration
	24	supine	weight-bearing single leg
	24	weight-bearing single leg	inward rotatory stress leg on platform - 3 kg
	24	weight-bearing single leg	inward rotatory stress - only muscle force
	24	weight-bearing single leg	outward rotatory stress leg on platform - 3 kg
	24	weight-bearing single leg	outward rotatory stress - only muscle force
	24	weight-bearing single leg	squatting
A multimodal assessment of cementless tibial baseplate fixation using radiography, radiostereometric analysis, and magnetic resonance imaging[25]	0.5	supine	weight-bearing single leg
	1.5	supine	weight-bearing single leg
	3	supine	weight-bearing single leg
	6	supine	weight-bearing single leg

Appendix Table 7. References

5. Ryd L, Lindstrand A, Rosenquist R, Selvik G. Tibial component fixation in knee arthroplasty. Clin Orthop Relat Res1986; (213): 141–9.

14. Broberg JS, Vasarhelyi EM, Lanting BA, et al. Migration and Inducible Displacement of the Bicruciate-Stabilized Total Knee Arthroplasty: A Randomized Controlled Trial of Gap Balancing and Measured Resection Techniques. J Arthroplasty 2022; 37(2): 252–8.

15. Laende EK, Richardson CG, Meldrum AR, Dunbar MJ. Tibial Component Migration After Total Knee Arthroplasty With High-Viscosity Bone Cement. J Arthroplasty 2021; 36(6): 2000–5.

16. Laende EK, Richardson CG, Dunbar MJ. Predictive value of short-term migration in determining long-term stable fixation in cemented and cementless total knee arthroplasties. Bone Joint J 2019; 101-B(7_Supple_C): 55–60.

17. Horsager K, Kaptein BL, Rømer L, et al. Dynamic RSA for the evaluation of inducible micromotion of Oxford UKA during step-up and step-down motion. Acta Orthop 2017; 88(3): 275–81.

18. Wilson DAJ, Astephen JL, Hennigar AW, Dunbar MJ. Inducible displacement of a trabecular metal tibial monoblock component. J Arthroplasty 2010; 25(6): 893–900.

19. Hansson U, Toksvig-Larsen S, Jorn LP, Ryd L. Mobile vs. fixed meniscal bearing in total knee replacement: a randomised radiostereometric study. The Knee 2005; 12(6): 414–8.

20. Bragonzoni L, Russo A, Loreti I, et al. The stress-inducible displacement detected through RSA in non-migrating UKR. The Knee 2005; 12(4): 301–6.

21. Regnér L, Carlsson L, Kärrholm J, Herberts P. Tibial component fixation in porous- and hydroxyapatite-coated total knee arthroplasty: a radiostereo metric evaluation of migration and inducible displacement after 5 years. J Arthroplasty 2000; 15(6): 681–9.

22. Puijk R, Koster LA, Pijls BGCW, et al. 5-year migration and inducible displacement of the uncemented LCS and ATTUNE rotating platform knee systems: a secondary report of a randomized controlled RSA trial. Acta Orthop 2025; 96: 59–65.

23. Hext RA, Kaptein BL, Howard JL, et al. Inducible displacement of cementless total knee arthroplasty components with conventional and weight-bearing CT-based radiostereometric analysis. J Orthop Res 2025; 43(3): 640–9.

24. Turgeon TR, Vasarhelyi E, Howard J, et al. Randomized controlled trial comparing traditional versus enhanced-fixation designs of a novel cemented total knee arthroplasty tibial component. Bone Jt. Open 2024; 5(1): 20–7.

25. Broberg JS, Koff MF, Howard JL, et al. A multimodal assessment of cementless tibial baseplate fixation using radiography, radiostereometric analysis, and magnetic resonance imaging. J Orthop Res 2024; 42(1): 100–8.

26. Teeter MG, Broberg JS, Howard JL, Lanting BA. Axial and Sagittal Rotation of Cementless Tibial Baseplates Occurs in Bone Under Joint Loading. J Arthroplasty 2023; 38(6): 1166–71.

27. Petersen MM, Nielsen PT, Lebech A, et al. Preoperative bone mineral density of the proximal tibia and migration of the tibial component after uncemented total knee arthroplasty. J Arthroplasty 1999; 14(1): 77–81.

28. Hilding MB, Yuan X, Ryd L.. The stability of three different cementless tibial components. A randomized radiostereometric study in 45 knee arthroplasty patients. Acta Orthop Scand 1995; 66(1): 21–27.

29. Ryd L, Carlsson L, Herberts P. Micromotion of a noncemented tibial component with screw fixation. An in vivo roentgen stereophotogrammetric study of the Miller-Galante prosthesis. Clin Orthop Relat Res 1993 ; (295): 218–25.

30. Ryd L, Lindstrand A, Stenström A, Selvik G. The influence of metal backing in unicompartmental tibial component fixation. An in vivo roentgen stereophotogrammetric analysis of micromotion. Arch Orthop Trauma Surg 1992; 111(3): 148–54.

31. Ryd L, Albrektsson BE, Herberts P, et al. Micromotion of noncemented Freeman-Samuelson knee prostheses in gonarthrosis. A roentgen-stereophotogrammetric analysis of eight successful cases. Clin Orthop Relat Res 1988; (229): 20512.

32. Ryd L, Lindstrand A, Rosenquist R, Selvik G. Micromotion of conventionally cemented all-polyethylene tibial components in total knee replacements. A roentgen stereophotogrammetric analysis of migration and inducible displacement. Arch Orthop Trauma Surg 1987; 106(2): 82–8.

33. Lam Tin Cheung K, Lanting BA, McCalden RW, et al. Inducible displacement of cemented tibial components ten years after total knee arthroplasty. Bone Joint J 2018; 100-B(2): 170–5.

34. Laende EK, Richardson CG, Dunbar MJ.. A randomized controlled trial of tibial component migration with kinematic alignment using patient-specific instrumentation versus mechanical alignment using computer-assisted surgery in total knee arthroplasty. Bone Joint J 2019, 101-B(8): 929–40.

35. Uvehammer J, Kärrholm J. Inducible displacements of cemented tibial components during weight-bearing and knee extension. Observations during dynamic radiostereometry related to joint positions and 2 years history of migration in 16 TKR. J Orthop Res 2001; 19(6):1168–77.
